# ATO alleviates T-cell immunosuppression by inducing apoptosis-associated secretion of IL-2 in FLT3-ITD-mutated acute myeloid leukemia

**DOI:** 10.1016/j.tranon.2026.102894

**Published:** 2026-07-20

**Authors:** Yiqing Ou, Guojun Hao, Yan Wu, Ziwen Guo, Xin He, Xiaomin Niu

**Affiliations:** a Department of Blood Transfusion, Zhongshan City People's Hospital, Zhongshan, Guangdong, 528403, China; b Department of Nephrology, Blood Puriﬁcation Center, Zhongshan City People's Hospital, Zhongshan, 528403, China; c Department of Intensive Care Unit, The Yancheng Clinical College of Xuzhou Medical University, The First people's Hospital of Yancheng, Yancheng , Jiangsu 224000, China; d Department of Hematology, Zhongshan City People's Hospital, Zhongshan, Guangdong, 528403, China

**Keywords:** Arsenic trioxide, Immunosuppression, Apoptosis, FLT3-ITD, Acute myeloid leukemia

## Abstract

**Background:**

Arsenic trioxide (ATO) is effective against FLT3-mutant acute promyelocytic leukemia (APL), but its role in non-APL FLT3-ITD-mutant acute myeloid leukemia (AML) remains unclear. This study aims to investigate the mechanistic impact of ATO on FLT3-ITD-mutated AML cells.

**Methods:**

This study utilized the CCK-8 assay, Wright–Giemsa staining, flow cytometry, Western blotting, and colorimetric assays to assess the effects of ATO on cell viability, cell morphology, apoptosis, Ki67 expression, reactive oxygen species (ROS), IL-2 levels, expression of apoptosis-related proteins, and LDH release in MV4–11 and HL-60 cells. Further validation was performed using NAC, PD98059 and LM22B-10. Following T-cell co-culture experiments, interferon-γ (IFN-γ) and granzyme B (GZMB) levels were measured. Differences in IL-2 expression were validated in primary cells from patients with AML.

**Results:**

Compared to HL-60 cells, ATO-treated FLT3-ITD-mutated MV4–11 cells exhibited lower IC50, reduced Ki67 expression, and elevated LDH levels. ATO induced dose- and time-dependent apoptosis in MV4–11 cells, accompanied by increased Cleaved Caspase-3 expression and Bax/Bcl-2 ratio. NAC attenuated ATO-induced ROS accumulation and apoptosis. ATO suppressed extracellular signal-regulated kinase (ERK) phosphorylation in a concentration-dependent manner, which was reversed by LM22B-10 but exacerbated by PD98059. Additionally, ATO enhanced IL-2 secretion in MV4–11 cells, and their conditioned medium elevated IFN-γ and GZMB levels in CD4^+^ and CD8^+^ T cells. Similar IL-2 upregulation was observed in primary cells from FLT3-ITD+ AML patients.

**Conclusions:**

ATO triggers apoptosis in FLT3-ITD-mutated AML cells via ROS/ERK signaling and modulates T-cell activity through IL-2 secretion, suggesting a dual role in tumor suppression and immune regulation.

**Significance:**

This study confirms that ATO is a potential candidate drug for the treatment of FLT3-ITD-mutant AML and provides mechanistic insights into its cytotoxic and immunomodulatory functions.

## Highlights

• Arsenic trioxide induces apoptosis via ROS/ERK signaling pathway in acute myeloid leukemia.
• Arsenic trioxide modulates T-cell activity through IL-2 secretion.
• Dual therapeutic mechanism is more marked in FLT3-ITD mutant acute myeloid leukaemia.

## Graphical abstract

**Figure:**
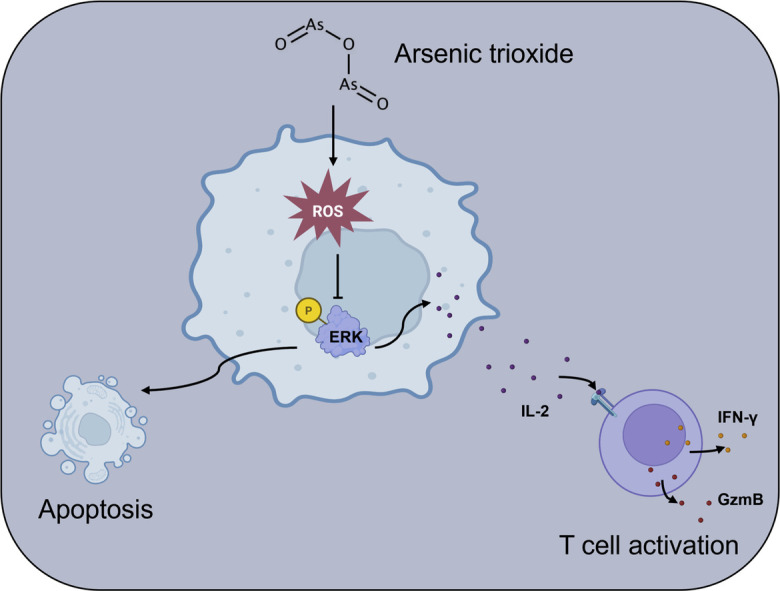

## Introduction

Acute myeloid leukemia (AML) is the most common acute myeloid malignancy in adults [1]. Epidemiologic studies demonstrate a median diagnostic age of 68 years, with an approximate 5-year overall survival (OS) rate of 30%. Furthermore, the mutation of the FMS-like tyrosine kinase 3 (FLT3) gene is present in approximately 30% of de novo AML cases [2] and is frequently overexpressed across hematopoietic malignancies. Notably, FLT3 activating mutations (FLT3mut) primarily occur in two domains: internal tandem duplications in the juxta membrane domain (FLT3-ITD) or point mutations in the tyrosine kinase domain (FLT3-TKD) [3]. Clinically, FLT3-ITD mutation has been incorporated into genetic risk stratification systems per current AML treatment guideline [4]. Patients with AML carrying FLT3-ITD have a higher relapse rate and worse clinical prognosis than patients without the mutation [[5], [6], [7]]*.* Although several FLT3 inhibitors have demonstrated clinical efficacy, substantial limitations remain. First-generation multi-kinase inhibitors often exhibit off-target effects leading to adverse events, while selective FLT3 inhibitors encounter acquired resistance and disease relapse [[8], [9], [10]]. Bruner et al. demonstrated that FLT3/ITD AML cells reactivate ERK within hours of FLT3 inhibitor exposure, and this resistance can be overcome by combining with a MEK inhibitor [11]. These therapeutic constraints underscore the critical need for novel agents that can effectively and safely target FLT3-ITD-mutated AML while overcoming current limitations of resistance and toxicity.

Notably, the FLT3-ITD mutation, a common genetic alteration in AML, has been linked to immunosuppressive mechanisms within the leukemic microenvironment. Bernstein et al. demonstrated that FLT3-ITD mutations in AML drive immune evasion by inducing a differentiation block in leukemic progenitors, thereby reducing immunogenicity [12].

Conventional anticancer agents have demonstrated notable immune-modulating effects alongside their direct cytotoxic activity. Decitabine, a first-line therapy for higher-risk myelodysplastic syndromes [13], has recently been shown to possess immunoregulatory properties. Emerging clinical data reveal that low-dose decitabine treatment can effectively modulate T cell homeostasis and restore immune tolerance in immune thrombocytopenia [14]. These findings highlight the dual therapeutic potential of conventional anticancer agents through both direct cytotoxic and immune-enhancing mechanisms. Arsenic trioxide (ATO) is an established agent in treating acute promyelocytic leukemia (APL). Recent studies have revealed the broad immunomodulatory of ATO in various cancer types. In hepatocellular carcinoma, ATO has been shown to promote immune activation by enhancing immunogenic cell death and activating the cGAS-STING-IFN signaling pathway [15], or by synergizing with B7H3-targeted immunotherapy to achieve tumor eradication [16]. Additionally, in pancreatic cancer models, low-dose ATO treatment was found to create a more favorable tumor microenvironment for immunotherapy [17]. These findings collectively demonstrate the multifaceted immunotherapeutic potential of ATO across different malignancies. Nevertheless, the immunoregulatory mechanisms through which ATO may suppress AML progression remain unclear.

The RAS/RAF/MEK/ERK (MAPK) signaling pathway plays a critical role in transmitting proliferation signals from extracellular stimuli to downstream effectors. Activation of this pathway leads to the phosphorylation of ERK1/2, which in turn regulates processes such as cell survival, proliferation, and differentiation [18]. Constitutive activation of the MAPK pathway is common in AML. Notably, FLT3-ITD is capable of activating the MAPK/ERK pathway [19]. A study by Khoury et al. demonstrated that the pan-RAF inhibitor LY3009120 induces apoptosis and inhibits proliferation in AML cells harboring RAS or FLT3 mutations by acting on the RAS/RAF/MEK/ERK and AKT/mTOR pathways [20]. Ricciardi et al. quantitatively analyzed p-ERK expression in 42 AML samples and found that p-ERK levels were significantly elevated in the majority of samples [21]. However, whether ATO exerts cytotoxic effects on FLT3-ITD-mutant AML cells via the MAPK pathway remains to be further elucidated.

In this study, we investigated the immunoregulation of ATO in AML patients harboring FLT3-ITD and showed that ATO exhibited selective growth inhibition and pro-apoptotic effects preferentially targeting FLT3-ITD-mutant AML cells over their wild-type counterparts. Importantly, we identified a novel immunomodulatory mechanism of ATO involving the restoration of T cell function through interleukin-2 (IL-2) secretion, with mechanistic studied revealing critical involvement of the ROS/ERK signaling pathway. These findings established ATO as a unique therapeutic agent with dual antileukemic and immune-enhancing properties, offering a promising strategy to improve treatment outcomes for FLT3-ITD-positive AML patients who typically showed poor response to conventional therapies.

## Materials and methods

### Cell lines and clinical samples

The HL-60 cell line was obtained from Newgainbio company. The MV4–11 cell line was obtained from Guangzhou Jennio Biotech. The MOLM13 cell line was obtained from Zhejiang Meisen Cell Technology Co., Ltd. Cells were maintained in Roswell Park Memorial Institute (RPMI) 1640 medium (Gibco) supplemented with 10% fetal bovine serum (FBS, Gibco). About 100 U penicillin and 100 μg streptomycin were added to the medium per milliliter (Beyotime, C0222). Bone marrow mononuclear cells (BMMCs) or peripheral blood mononuclear cells (PBMCs) were collected from treatment-naïve AML patients or healthy donors at the Zhongshan City People’s Hospital and using by Ficoll-Hypaque density gradient centrifugation. The clinical characteristics and laboratory features are summarized in Table 1, Table 2. The study was approved by Zhongshan City People’s Hospital Research Ethics Committee.

**Table 1:** Characteristics of healthy donors.

No.	Gender	Age	No.	Gender	Age
1	Female	38	6	Male	45
2	Female	53	7	Male	30
3	Female	28	8	Female	43
4	Male	60	9	Male	50
5	Female	56	10	Female	37

**Table 2:** Clinical features at initial presentation in 8 patients with acute myeloid leukaemia.

No.	Age	Gender	WBC(×10^9^/L)	Blasts (%)	FLT3 status	Mutant(%)	Sample
1	52	Female	403.58	82	ITD+	30.15	BM
2	53	Male	31.16	88	ITD+	37.47	BM
3	34	Male	94.42	91.4	ITD+	51.62	BM
4	61	Female	47.39	80.5	ITD+	25.13	BM
5	30	Female	105.06	92	WT	-	BM
6	68	Male	519.97	80	WT	-	BM
7	66	Female	63.12	94	WT	-	BM
8	60	Female	70.42	60	WT	-	BM

**BM**, bone marrow; **FLT3**,Fms-like tyrosine kinase 3; **ITD+,** internal tandem duplication positive; **WT,** wild-type; **WBC,** white blood cell. FLT3 status was classified as FLT3-ITD+ or WT.

### Wright-Giemsa staining and lactate dehydrogenase (LDH) assays

Cells were treated with or without ATO (10 μM) for 12 h. Then cells were collected and the precipitate was applied to slides. The slides were subjected to Wright-Giemsa staining and then observed or photographed under a microscope. Cells were treated with ATO (2.5, 5, 10 µM) for 12 h, followed by detection of LDH release rate (Roche).

### CCK8 analysis

Cell viability was measured by CCK8 method. HL-60(1 × 10^6^ cells/well) and MV4–11(5 × 10^6^ cells/well) in the logarithmic phase were seeded into 96-well plates simultaneously with various concentrations of different compounds (diluted in saline solution, final concentration of 0.625–20 µM) or vehicle for 12, 24 and 48 h. Then cultured with 10 μl CCK-8 (Dojindo, CK04) for 4 h. The absorbance was determined at 450 nm. The half-maximal inhibitory concentration (IC50) value was calculated using GraphPad Prism software.

### Apoptosis and reactive oxygen species(ROS) assays

Annexin V-FITC/PI Kit (BD Biosciences, 559,763) was employed for apoptosis analysis. Cells were seeded in 6-well plates, treated with ATO, N-acetyl-l-cysteine (NAC, MCE HY-B0215) or pretreatment with NAC, PD98059 (MCE HY-104,047) or LM22B-10 (MCE HY-12,028) before treatment with ATO with different concentration for indicated time. Cells were washed twice with cold phosphate-buffered saline (PBS) and resuspended in 500 µl of Binding Buffer plus Annexin V-FITC and propidium iodide (PI), and then cells were subjected to flow cytometry analysis by BD FACSCanto instrument. Compensation was performed using single-stained controls (Supplementary Fig. 1). For ROS analysis, a 2,7-dichlorodihydrofluorescein diacetate (DCFH-DA) fluorescent probe (KGA7502–5, KeyGEN BioTECH) was used according to the instructions. Cells treated with ATO, NAC or pretreatment with 5 mM NAC for 2 h before treatment with ATO and washed twice with PBS and incubated in the serum-free medium containing 5 μM DCFH-DA for 20 min at 37 °C in the dark. Finally, cells were collected for analysis by the flow cytometer. Data were analyzed with FlowJo software.

### Proliferation marker protein *(Ki67)* and IL-2 analysis

Cells were treated with ATO, NAC or pretreated with NAC, PD98059 or LM22B-10 before treatment with ATO with different concentration for indicated time. Cells were fixed and permeabilized by using eBioscience™ FOXP3/Transcription Factor Staining Buffer Set (Thermo Fisher Scientific, 00–5523–00) for 60 min. Then cells incubated with APC anti-human Ki67 (Biolegend, 350,514) for 30 min on ice for Ki67 detection. Whereas IL-2 was determined by incubation with IL-2 antibody.

### Western blot

To prepare protein samples, 3 × 10^6^ cells of each sample were collected and washed by PBS twice. Each sample was lysed in RIPA buffer (Beyotime, P0013B) with a protease inhibitor cocktail (Beyotime, P1081) and boiled at 100 °C. Then protein samples were separated by SDS-PAGE and transferred to a PVDF membrane. The antibodies and the usage concentrations are as follows: anti-p-ERK (ab201015, WB, 1:1000), anti-ERK (ab184699, WB, 1:10,000),anti-Caspase3(Zenbio R23315, 1:1000), anti-Cleaved Caspase-3(Zenbio R23727, 1:1000), anti-Bax(Zenbio R22708, 1:1000), anti-Bcl2(Zenbio R22494, 1:1000), β-Tubulin (Beyotime, AF1216, 1:5000).

### qPCR assay

The total RNA of each cell type was isolated by TRIzol reagent (Invitrogen, 15596018). cDNA synthesis was performed with Thermo Scientific RevertAid RT Kit (Thermo Fisher Scientific, K1622). qPCR was conducted using Roche LightCycler 480 with a ChamQ SYBR qPCR Master Mix (Vazyme, Q311–02). The primer sequences were IL-2 F: 5′-AGAACTCAAACCTCTGGAGGAAG-3′, IL-2 R: 5′-GCTGTCTCATCAGCATATTCACA-3′.

### Plasmid construction and lentivirus infection

IL2 knock-down plasmid was constructed by ligating shRNA to PLKO.1-TRC vector. The shRNA sequences used for IL2 knock-down were shIL2–1: 5′-CCAGGGACTTAATCAGCAATA-3′, shIL2–2: 5′-GCATCATCTCAACACTGACTT-3′. 293FT cells were co-transfected with IL2 knock-down plasmid and packaging vectors pMD2.G and pXPAS2 using Lipofectamine 2000 transfection reagent (Invitrogen, 11,668–019). Lentivirus was collected 48 h after transfection followed by cell infection.

### Cell cycle analysis

Cell cycle analysis was performed by flow cytometry. Briefly, cells were collected, washed with PBS, and fixed in 70% ethanol at 4 °C for overnight. Fixed cells were washed followed by staining with propidium iodide (50 µg/mL) in the presence of RNase A (100 µg/mL) for 30 min at 37 °C in the dark, with apoptosis, G0/G1, S, and G2/M phase percentages determined using ModFit LT software.

### T cell coculture assay

In a co-culture experiment involving AML cells and T cells, 5 × 10⁵ AML cells were cultured in RPMI 1640 medium containing 10% FBS and divided into a blank control group and a 2.5 μM ATO-treated group, and cultured for 24 h. The cell supernatants were then collected and centrifuged to obtain the control culture medium and the ATO-treated culture medium, respectively. PBMCs were isolated from healthy volunteers using the Ficoll-Hypaque density gradient centrifugation method and transferred to a 96-well plate for 24-hour culture. The 96-well plates used in this experiment were pre-coated overnight at 4 °C with 2.5 μg/mL anti-human cluster of differentiation 3 (CD3) antibody (Biolegend, 317326). The 0.5 μg/mL anti-human cluster of differentiation 28 (CD28) antibody (Biolegend, 302934) was added upon seeding the cells. The experiment comprised five groups: Blank group (200 μL RPMI 1640), Alone group (200 μL RPMI 1640), Activation group (200 μL RPMI 1640 + anti-CD3/CD28), Control group (200 μL control medium + anti-CD3/CD28), and 2.5 μM ATO group (200 μL ATO-treated medium + anti-CD3/CD28). T cells were then collected and analyzed by flow cytometry.

### Statistical analysis

Statistical data were analyzed using GraphPad Prism (10.2.0). The comparison between the two groups was conducted using the Student’s *t*-test and the comparison between multiple groups was performed using one-way and two-way analysis of variance (ANOVA) followed by Sidak’s multiple comparison test. For clinical samples (n = 4 patients per group): Mann-Whitney U test for between-group comparisons and Friedman test for within-group comparisons. Data represented as means ± standard deviation (SD) of at least n = 3 independent experiments. The *P* value was set as statistical significance including *P < 0.05; ** *P* < 0.01; *** *P* < 0.001.

## Results

### ATO inhibits the proliferation and induces apoptosis in FLT3-ITD-positive AML cells

**Fig. 1:**
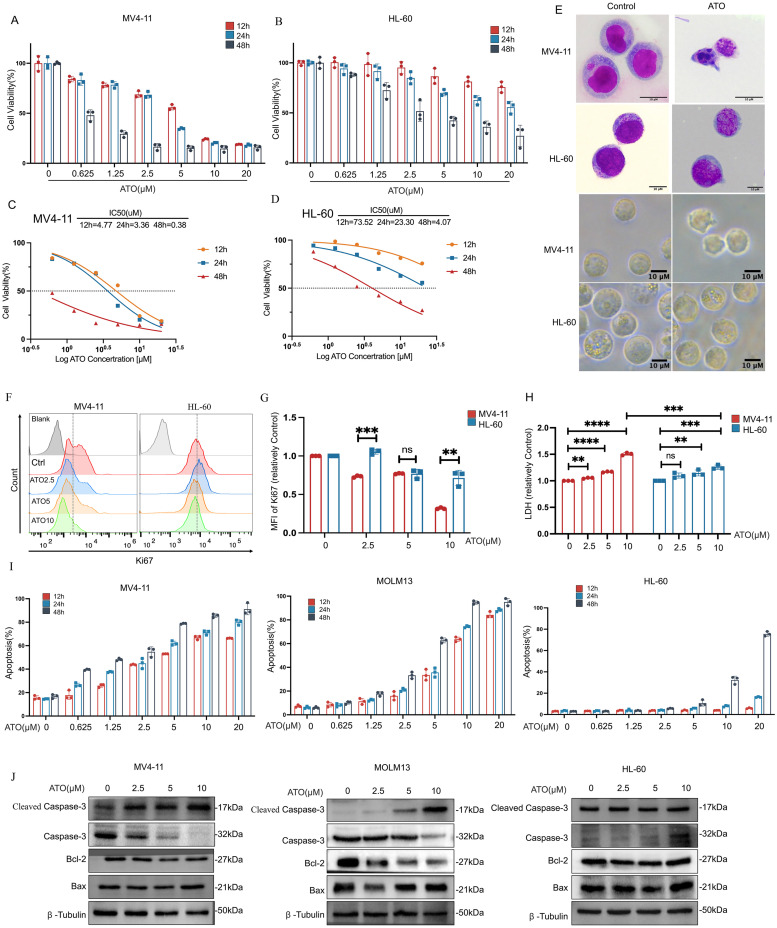
ATO inhibits the proliferation and induces apoptosis in FLT3-ITD positive cells. MV4–11, MOLM13 and HL-60 cells were challenged with increasing doses of ATO (0, 0.625, 1.25, 2.5, 5, 10, 20 µM) for 12, 24 and 48 h. MV4–11 and HL-60 cell viability (A, B) was determined by the CCK8 assay (n = 3). IC50 values (C, D) were calculated. Apoptosis of MV4–11, MOLM13 and HL-60 cells was detected by Annexin V-FITC/PI staining (I) (n = 3). Morphological changes in MV4–11 and HL-60 cells following ATO treatment (10 µM, 12 h) were observed using Wright–Giemsa staining and live-cell imaging (scale bar = 10 µm) (E). Ki-67 assay (F, G) and LDH release detection (H) were performed on MV4–11 and HL-60 cells treated with different doses of ATO (0, 2.5, 5, 10 µM) for 12 h (n = 3). Apoptosis-related proteins were measured in MV4–11, MOLM13 and HL-60 cells after treatment with 2.5, 5 and 10 μM ATO for 12 h (J). β-Tubulin was used as an internal control. Data are presented as mean ± SD. *p < 0.05, **p < 0.01, ***p < 0.001. MFI: mean fluorescence intensity.

To evaluate the cytotoxic effect of ATO, we exposed FLT3-ITD-positive MV4–11 and FLT3-ITD-negative HL-60 cells to increasing concentrations of ATO and assessed cell viability. As shown in Fig. 1A and B, ATO exposure resulted in a dose- and time-dependent reduction in viability in both cell lines following 12 h, 24 h, and 48 h incubations. MV4–11 cells exhibited a more rapid decline in viability and a lower IC50 compared to HL-60 cells, suggesting FLT3-ITD-positive AML cells are particularly vulnerable to ATO-mediated cytotoxicity (Fig. 1C, D). Consistent with these observations, Wright-Giemsa staining and phase-contrast images demonstrated characteristic apoptotic morphology in MV4–11 cells, including cellular shrinkage and nuclear fragmentation, after 12 h treatment with 10 μM ATO. In contrast, HL-60 cells retained normal cellular architecture under identical treatment conditions (Fig. 1E). To further characterize the distinct cellular responses to ATO in AML cells, we assessed cell proliferation and cytotoxicity by detecting Ki67 expression and LDH release, respectively. Consistent with the viability data, ATO-exposed MV4–11 cells displayed substantially reduced Ki67 expression (Fig. 1F, G). Additionally, MV4–11 released higher levels of LDH than HL-60 after 12 h exposure to 10 μM ATO (Fig. 1H), which confirmed preferential cytotoxicity of ATO in FLT3-ITD-positive AML.

To elucidate the mechanism underlying ATO-mediated growth inhibition, we quantitatively analyzed apoptosis in FLT3-ITD-positive cells (MV4–11 and MOLM13) and FLT3-ITD-negative cells (HL-60) following ATO exposure. As shown in Fig. 1I and Supplementary Fig. 2, ATO exposure induced significant apoptosis in MV4–11 and MOLM13 cells, with observable effects after 12-hour treatment at 1.25 μM and exhibited marked dose-dependent and time-dependent characteristics. Furthermore, we examined the cell cycle in MV4–11 and MOLM13 cells. The results (Supplementary Fig. 3) showed that apoptotic cells, characterised by DNA fragmentation, exhibited time- and concentration-dependent changes, whilst no corresponding alterations were observed in the G0/G1, S or G2 phases, thereby further confirming the occurrence of ATO-induced apoptosis. To further confirm that ATO-induced growth inhibition was accompanied with apoptosis, we evaluated the expression of apoptotic proteins. ATO treatment resulted in both concentration-dependent elevation of Cleaved Caspase-3 levels and a significant increase in the Bax/Bcl-2 ratio (Fig. 1J), which further confirmed that ATO triggers apoptosis in FLT3-ITD-positive AML cells through activation of caspase-dependent apoptotic pathways.

### ATO induces apoptosis through ROS-mediated damage in FLT3-ITD-positive AML cells

**Fig. 2:**
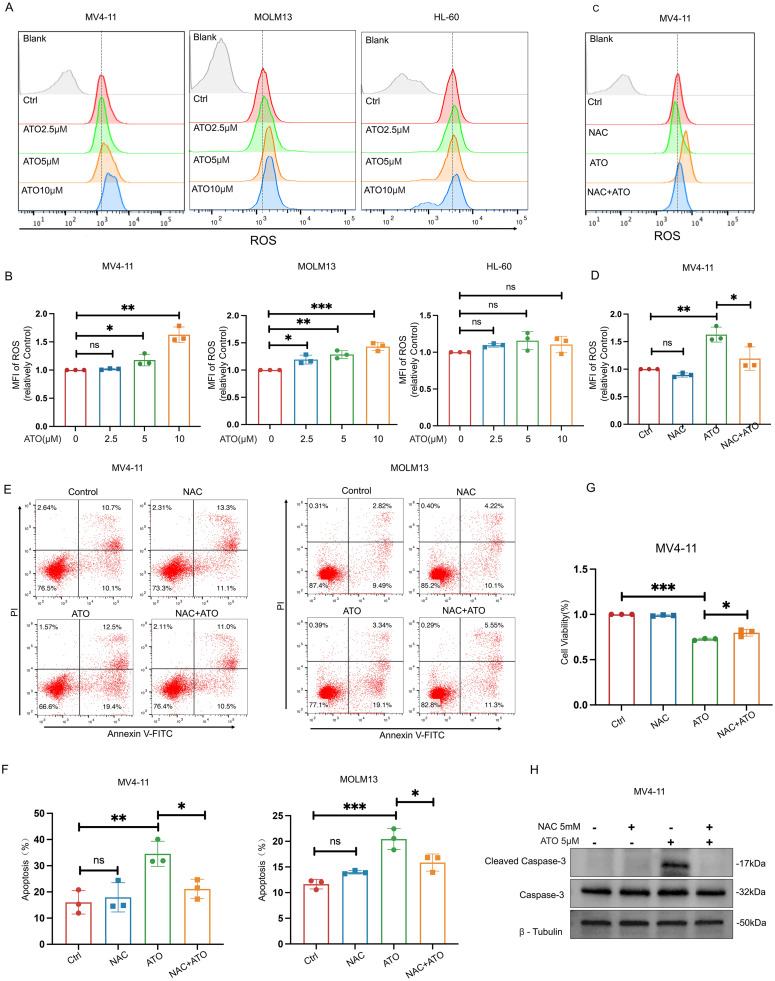
ATO induces apoptosis through ROS-mediated damage in FLT3-ITD positive cells. MV4–11, MOLM13 and HL-60 were treated with 2.5, 5 and 10 μM ATO for 6 h and intracellular ROS were measured using DCFH-DA probe (A). Quantitative analysis of DCFH-DA MFI (B) (n = 3). In subsequent experiments, MV4–11 and MOLM13 cells were divided into four groups: pretreatment with 5 mM NAC for 2 h followed by 10 μM ATO for 6 h; NAC (5 mM) alone; ATO (10 μM) alone; untreated control. Pretreatment with 5 mM NAC for 2 h partially reversed ROS production in MV4–11 cells (C, D) (n = 3). Apoptosis in MV4–11 and MOLM13 cells (E, F) and cell viability in MV4–11 cells (G) were assessed using the Annexin V/PI assay and the CCK-8 assay, respectively (n = 3). Protein expression of Cleaved Caspase-3 and Caspase-3 in MV4–11 cells lysate (H). β-Tubulin was used as an internal control. Data are presented as mean ± SD. *p < 0.05, **p < 0.01, ***p < 0.001. ns, no statistical significance.

To determine whether oxidative stress contributes to ATO-induced apoptosis in FLT3-ITD-positive AML cells, we measured intracellular ROS levels. ATO induced a significant increase of intracellular ROS in FLT3-ITD-positive cells MV4–11 and MOLM13 after incubation with 5 μM ATO for 6 h, while no significant changes were observed in FLT3-ITD-negative cells HL-60 (Fig. 2A, B). To determine the causality between ROS accumulation and cell death, we employed the ROS scavenger NAC and noticed that pretreatment with 5 mM NAC for 2 h effectively attenuated ATO-induced ROS generation in MV4–11 (Fig. 2C and D). Subsequent analyses demonstrated that NAC pretreatment significantly restored cell viability of MV4–11 (Fig. 2G) and reduced apoptotic rates of MV4–11 and MOLM13 (Fig. 2E, Fig. 2, Fig. 2). These findings demonstrate that ATO triggers apoptosis in FLT3-ITD-positive AML cells through a ROS-dependent mechanism, as evidenced by the reversal of cytotoxic effects upon antioxidant intervention.

### ROS/ERK signaling mediated ATO-Induced apoptosis in FLT3-ITD-positive AML cells

**Fig. 3:**
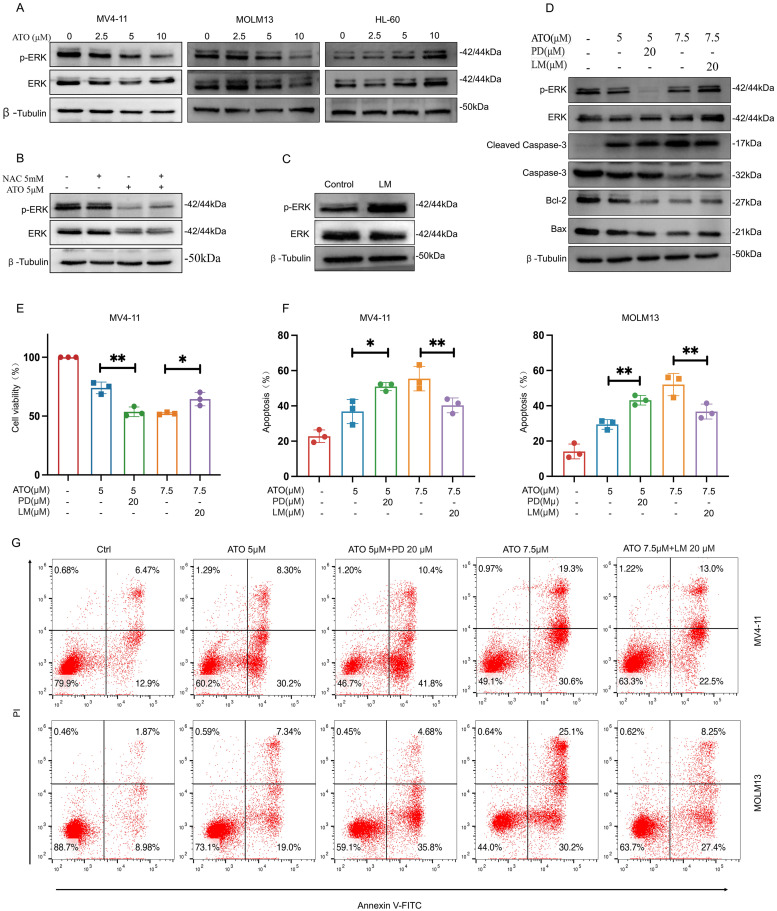
ROS/ERK signaling mediates ATO effect in FLT3-ITD positive cells. The protein expression of p-ERK and ERK was observed by western blot in MV4–11, HL-60 and MOLM13 cells treated with 2.5, 5, and 10 µM ATO for 12 h (A). The protein levels of p-ERK and ERK were examined in MV4–11 cells lysates after 12 h of treatment with 5 µM ATO, with or without pre-treatment with 5 mM NAC (B). MV4–11 cells were treated with the ERK agonist LM22B-10 (LM) 20 μM for 18 h. Expression levels of p-ERK and ERK were determined by Western blot (C). MV4–11 cells were pretreated with the LM22B-10 20 μM or ERK inhibitor PD98059 (PD) 20 μM for 6 h, followed by the ATO treatment for 12 h. Expression levels of p-ERK, ERK, Cleaved Caspase-3, Caspase-3, Bcl-2 and Bax in MV4–11 cells were determined by Western blot (D). Cell viability of MV4–11 (E) and apoptosis in MV4–11 and MOLM13 (F, G) were assessed using the CCK-8 assay and Annexin V-FITC/PI double staining, respectively (n = 3). β-Tubulin was used as an internal control. Data are presented as mean ± SD. *p < 0.05, **p < 0.01.

To investigate whether ERK participated in ATO-induced apoptosis, we analyzed ERK1/2 phosphorylation status in AML cells after exposure to ATO. As shown in Fig.3A, ATO treatment induced concentration-dependent dephosphorylation of ERK1/2 in FLT3-ITD-positive cells MV4–11 and MOLM13, with no corresponding modulation observed in FLT3-ITD-negative cells HL-60, which suggested the FLT3-ITD-dependent regulation of ERK1/2 phosphorylation by ATO, prompting our selection of MV4–11 cells for subsequent mechanistic investigations. Consistently, the ROS scavenger NAC completely abrogated ATO-mediated ERK inactivation (Fig. 3B), demonstrating that ATO induces ERK dephosphorylation through ROS generation.

To functionally validate the role of ERK in ATO-induced apoptosis, we employed pharmacological modulation using the ERK agonist LM22B-10 and inhibitor PD98059. To verify the effect of LM22B-10, we treated cells with LM22B-10 and observed that it significantly activated ERK phosphorylation in MV4–11 cells (Fig. 3C). Then pretreatment with 20 μM LM22B-10 for 6 h could effectively restore ERK phosphorylation in ATO-exposed MV4–11 cells, while PD98059 pretreatment further enhanced this inhibitory effect (Fig. 3D). Likewise, LM22B-10 pretreatment attenuated ATO-mediated apoptotic responses, as evidenced by reduced Cleaved Caspase-3 expression and decreased Bax/Bcl-2 ratio. In contrast, PD98059 pretreatment exacerbated the expression of apoptotic proteins (Fig. 3D). Furthermore, assessment of cell viability and apoptosis rates revealed that pretreatment with 20 μM LM22B-10 for 6 h partially reversed ATO-induced cytotoxicity, characterized by increased viability and decreased apoptosis (Fig. 3E–G). Conversely, 20 μM PD98059 pretreatment for 6 h increased apoptosis and decreased viability in ATO-exposed MV4–11 cells (Fig. 3E–G). These results collectively demonstrate that ATO triggers oxidative stress-mediated inhibition of ERK signaling, which subsequently leads to suppressed proliferation and enhanced apoptosis in FLT3-ITD-positive AML cells.

### ATO-modulated IL-2 expression in FLT3-ITD-positive AML cells is associated with apoptosis

**Fig. 4:**
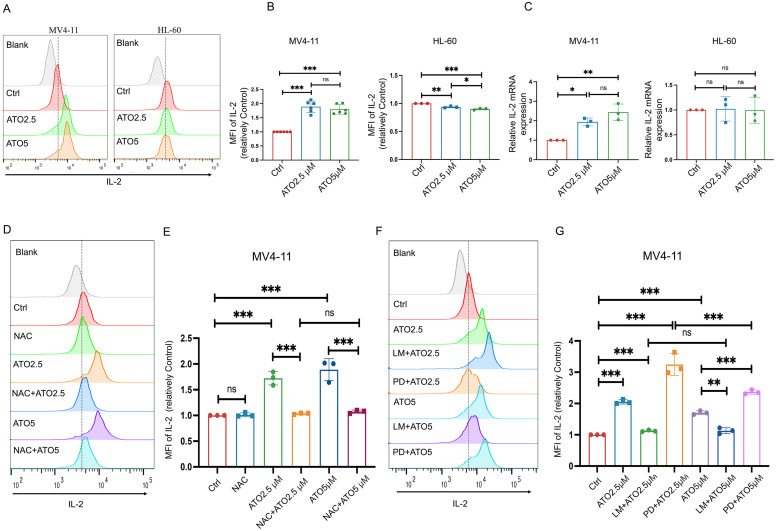
IL-2 correlates with ATO induction of apoptosis in FLT3-ITD positive cells. IL-2 expression levels were assessed by flow cytometry (A and B) and RT-qPCR (C) in MV4–11 and HL-60 cells following treatment with 0, 2.5 and 5 µM ATO for 12 h (n = 3). MV4–11 cells were pretreated with LM22B-10,PD98059 or NAC followed by 2.5 µM and 5 µM ATO for 12 h. IL-2 expression (D, F) and MFI (E, G) were measured (n = 3). Data are presented as mean ± SD. *p < 0.05, **p < 0.01, ***p < 0.001. ns, no statistical significance.

To explore the relationship between ATO-induced apoptosis and IL-2 expression in FLT3-ITD-positive AML cells, we examined IL-2 expression patterns in both FLT3-ITD-positive and negative AML cells following ATO exposure. Studies have demonstrated that apoptosis can trigger the release of cytokines, such as IL-1α, which may modulate the immune microenvironment and influence tumor progression [22]. Notably, hematologic malignancies demonstrate distinct patterns of aberrant IL-2 expression compared to solid tumors [23,24]. Our results show that IL-2 expression was significantly increased in ATO-treated FLT3-ITD-positive MV4–11 cells, whereas no such increase was observed in FLT3-ITD-negative HL-60 cells (Fig. 4A–C). This differential response suggests that ATO preferentially induces IL-2 expression in FLT3-ITD-positive AML cells. To further elucidate the potential association between IL-2 expression and apoptotic responses following ATO treatment, we performed pharmacological intervention studies targeting apoptosis in ATO-exposed AML cells. As shown in Fig. 4D–G, pharmacological inhibition with NAC dose-dependently attenuated ATO-induced IL-2 expression (Fig. 4D, E), paralleling the corresponding decrease in apoptosis (Fig. 2E, F). Consistently, modulation of apoptosis by using the specific agonist LM22B-10 and inhibitor PD98059 of ERK pathway (Fig. 3E, F) also demonstrated corresponding effects on IL-2 regulation (Fig. 4F, G). These results indicate that ATO modulates IL-2 expression in FLT3-ITD-positive AML cells is associated with apoptosis.

### ATO alleviates immunosuppression of T cells by inducing apoptosis-associated secretion of IL-2

**Fig. 5:**
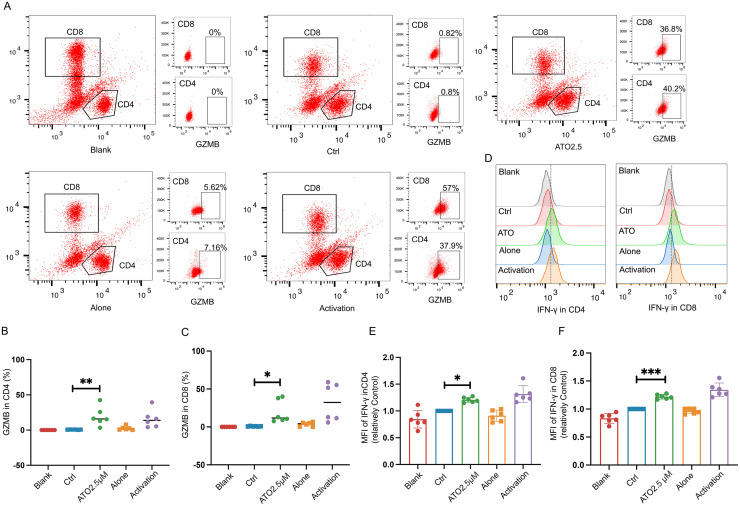
ATO alleviates T cell immunosuppression. The conditioned medium from MV4–11 cells treated with 2.5 µM ATO for 24 h was co-cultured with T cells. Expression of GZMB and IFN-γ in CD4^+^ and CD8^+^ T cells was detected (A, D) and quantitatively analyzed (B, C and E, F) (n = 6). Data are presented as mean ± SD. *p < 0.05, **p < 0.01, ***p < 0.001.

**Fig. 6:**
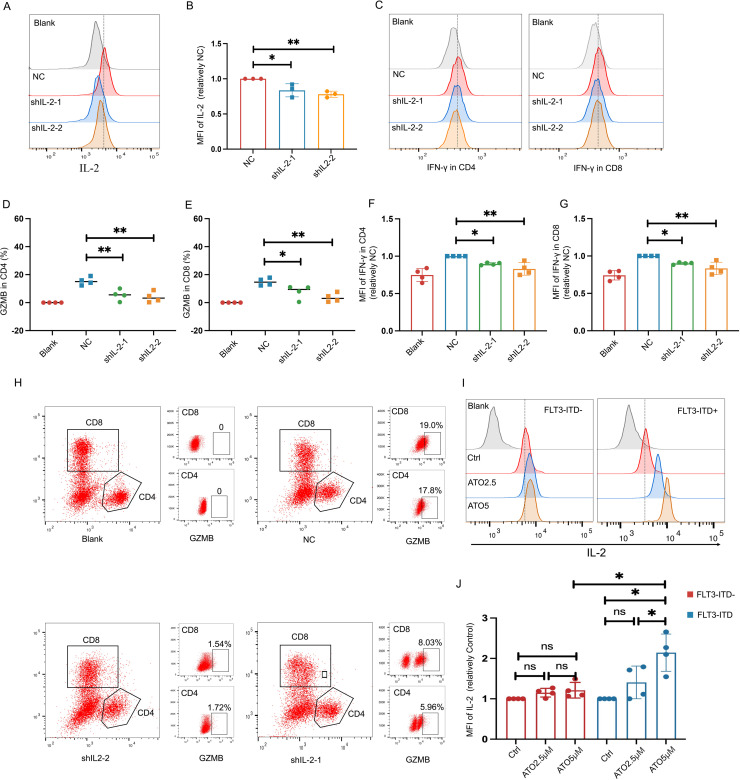
ATO alleviates immunosuppression via the IL-2 signaling pathway. IL-2 shRNA was transfected into MV4–11 cells. The cells were harvested to detect IL-2 expression by flow cytometry (A). Quantification of IL-2 MFI levels (B) (n = 3). The conditioned medium from IL-2-deficient MV4–11 cells treated with 2.5 µM ATO for 24 h was co-cultured with T cells. Expression of GZMB and IFN-γ in CD4^+^ and CD8^+^ T cells was detected (C,H) and quantitatively analyzed (D-G) (n = 4). BMMCs were isolated from FLT3-ITD+ (n = 4) and FLT3-ITD- (n = 4) patients. IL-2 expression was analyzed after culturing cells with 2.5 µM or 5 µM ATO for 12 h (I). Flow cytometry data quantification (J) (n = 4). Data are presented as mean ± SD. For statistical analysis, the Friedman test was used for within-group comparisons and the Mann-Whitney U test for between-group comparisons. NC, negative control cells transfected with negative control shRNA; shIL-2–1 and shIL-2–2, knockdown of MV4–11 cells transfected with IL-2 shRNA; FLT3-ITD+, FLT3-ITD positive; FLT3-ITD-, FLT3-ITD negative. *p < 0.05, **p < 0.01.

To determine whether ATO-induced IL-2 secretion from FLT3-ITD-positive AML cells enhances T-cell activation, we co-cultured T cells with conditioned medium from ATO-treated MV4–11 cells and measured granzyme B (GZMB) and interferon-γ (IFN-γ) expression. IL-2 is a pleiotropic cytokine that exerts both immunostimulatory and immunosuppressive effects through diverse immune cell populations. While IL-2 signaling promotes T-cell proliferation and enhances effector functions [25], its role in the tumor microenvironment remains complex. Our results show that ATO-treated MV4–11 cells significantly upregulated GZMB expression in both CD4^+^ and CD8^+^ T cells, indicating enhanced cytotoxic potential (Fig. 5A–C and Supplementary Fig. 4). Furthermore, we observed increased IFN-γ expression—a key marker of T-cell activation—in T cells co-cultured with ATO-exposed MV4–11 cells (Fig. 5D–F). These findings suggest that ATO might enhance T-cell activation in FLT3-ITD-positive AML by inducing IL-2 secretion. To validate this hypothesis, we generated IL-2 knockdown MV4–11 cells using shRNA (Fig. 6A,B). As expected, both GZMB and IFN-γ expression were significantly reduced in CD4^+^ and CD8^+^ T cells co-cultured with IL-2-deficient MV4–11 cells following ATO treatment (Fig. 6D–H). Furthermore, BMMCs from FLT3-ITD+ (n = 4) and FLT3-ITD- (n = 4) AML patients (clinical characteristics summarized in Table 2) were treated with 2.5 µM or 5 µM ATO for 12 h. ATO treatment specifically elevated IL-2 levels in FLT3-ITD+ but not FLT3-ITD- patient samples (Fig. 6I, J). These results collectively demonstrate that ATO enhances T-cell activation through IL-2 induction specifically in FLT3-ITD-positive AML.

## Discussion

The FLT3-ITD mutation represents one of the most clinically significant genetic alterations in acute myeloid leukemia, consistently associated with poor prognosis in terms of both relapse-free and overall survival [2]. Our current study provides compelling evidence that ATO exerts dual therapeutic effects against FLT3-ITD-mutated AML through direct cytotoxic activity and immunomodulation, offering a promising approach to overcome the limitations of current treatment strategies for this high-risk patient population.

The differential sensitivity of FLT3-ITD-mutated AML cells to ATO forms the foundation of its therapeutic potential. While ATO demonstrated cytotoxic effects against both FLT3-ITD-mutated (MV4–11) and wild-type (HL-60) cell lines, the magnitude of apoptosis induction and proliferation inhibition was markedly more pronounced in FLT3-ITD-positive cells. This observation aligns with emerging clinical evidence suggesting that ATO-containing regimens may mitigate the negative prognostic impact of FLT3 mutations in acute promyelocytic leukemia [[26], [27], [28]]. Importantly, the effective ATO concentrations used in this study (2.5–5 µM) fall within the range of clinically achievable peak plasma concentrations (5.54–7.30 µM) [29]. These observations provide mechanistic insights into this phenomenon through rigorous in vitro experimentation.

At the molecular level, we have elucidated a coherent ROS/ERK signaling axis that mediates ATO's selective cytotoxicity in FLT3-ITD-mutated AML. Reactive oxygen species (ROS), well-established as crucial mediators of drug-induced apoptosis [30,31], were significantly elevated following ATO treatment in MV4–11 cells. The complete reversal of ATO's effects by the antioxidant NAC confirms ROS as central to this cytotoxic mechanism. Importantly, we established a direct link between ATO-induced ROS generation and suppression of ERK phosphorylation, a critical regulator of cellular proliferation and survival [32]. Pharmacological modulation experiments using the ERK agonist LM22B-10 and inhibitor PD98059 provided functional validation of this pathway, demonstrating that ERK inactivation is both necessary and sufficient for ATO-mediated apoptosis in FLT3-ITD-mutated cells.

Beyond its direct antileukemic effects, our study reveals a novel immunomodulatory dimension of ATO's mechanism of action. In the context of AML's well-documented immunosuppressive microenvironment [33], we demonstrated that ATO treatment of FLT3-ITD-mutated cells triggers dose-dependent IL-2 secretion, resulting in significant enhancement of T-cell effector function as evidenced by increased IFN-γ and granzyme B expression. The specificity of this response to FLT3-ITD-mutated cells, confirmed in primary patient samples, suggests a mutation-dependent immunomodulatory mechanism that may relate to the distinct redox biology of FLT3-ITD-positive AML. This finding is particularly significant given the growing recognition of immunotherapy's transformative potential in cancer treatment [34], and the current paucity of effective immunotherapeutic options for AML.

**Fig. 7:**
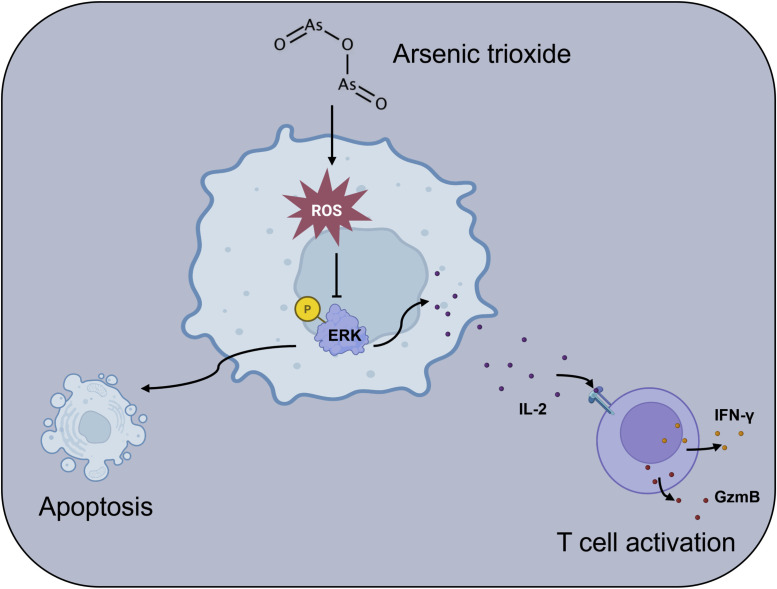
Mechanistic schematic of ATO in tumor suppression and immune regulation.

The convergence of these two mechanisms-direct cytotoxicity through ROS/ERK signaling and immune activation via IL-2 secretion - positions ATO as a unique therapeutic agent capable of simultaneously addressing both the malignant clone and its immunosuppressive microenvironment in FLT3-ITD-mutated AML. These mechanisms are illustrated in Fig. 7. This dual action may explain the clinical observations of ATO's efficacy in overcoming the poor prognosis associated with FLT3 mutations, while also suggesting potential synergies with emerging immunotherapeutic approaches. However, as this study is limited to in vitro experiments, the therapeutic value of ATO for FLT3 ITD-mutant AML requires further validation in vivo experiments. Furthermore, the number of patient-derived primary BMMCs needs to be increased in order to validate the effect of ATO on the IL-2 response of primary cells from FLT3 ITD-positive AML.

In conclusion, our findings establish ATO as a multifaceted therapeutic agent for FLT3-ITD-mutated AML, with mechanisms spanning from molecular pathway modulation to immune microenvironment reprogramming. The ROS/ERK/IL-2 axis identified in this study not only provides a mechanistic framework for understanding ATO's clinical efficacy but also opens new avenues for therapeutic optimization in this genetically defined, high-risk AML subset. These results support the continued investigation of ATO-containing regimens for FLT3-ITD-mutated AML and suggest potential combinations with immunotherapeutic agents to further enhance treatment outcomes.

## CRediT authorship contribution statement

**Yiqing Ou:** Writing – original draft, Validation, Investigation, Formal analysis, Data curation. **Guojun Hao:** Writing – review & editing, Validation, Investigation, Formal analysis, Data curation. **Yan Wu:** Writing – review & editing, Validation, Investigation, Formal analysis, Data curation. **Ziwen Guo:** Investigation. **Xin He:** Investigation. **Xiaomin Niu:** Writing – review & editing, Supervision, Project administration, Funding acquisition, Conceptualization.

## Declaration of competing interest

The authors declare the following financial interests/personal relationships which may be considered as potential competing interests:

Xiaomin Niu reports financial support was provided by Scientific research project of Traditional Chinese Medicine Bureau of Guangdong Province. Xiaomin Niu reports financial support was provided by Zhongshan Municipal Science and Technology Bureau. If there are other authors, they declare that they have no known competing financial interests or personal relationships that could have appeared to influence the work reported in this paper.

## Appendix Supplementary materials

Supplementary Figure 1. Compensation controls for Annexin V/PI staining in MV4–11, HL-60, and MOLM13 cells. Image, application 1

Supplementary Figure 2. The apoptotic effects of ATO on MV4–11, MOLM13, and HL-60 cells. MV4–11, MOLM13, and HL-60 cells were exposed to different concentrations of ATO (0, 0.625, 1.25, 2.5, 5, 10, 20 µM) for 12, 24, and 48 h, and apoptosis was detected using Annexin V-FITC/PI staining. Image, application 2

Supplementary Figure 3. The effect of ATO on the cell cycle of FLT3-ITD-positive cells. The effects of ATO on the Apoptosis, G0/G1, S and G2/M phases of MV4–11 and MOLM13 cells following treatment with different concentrations (0, 2.5, 5, 10 μM) for 12, 24 and 48 h. a: significant versus control, b: significant versus ATO 2.5 μM, c: significant versus ATO 5 μM. Image, application 3

Supplementary Figure 4. Typical gating strategies for CD4⁺/CD8⁺ T cell analysis. (A) Exclusion of debris using FSC‑A/SSC‑A; (B) Identification of single cells using FSC‑A/FSC‑H; (C) Selection of live cells using live/dead staining; (D) Definition of CD4⁺ and CD8⁺ T cell subsets using a CD4 versus CD8 plot; (E) Measurement of GZMB and IFN-γ levels in CD8⁺ T cells; (F) Measurement of GZMB and IFN-γ levels in CD4⁺ T cells. Image, application 4

Image, application 5
