# Supplementary figures and images for: ATO alleviates T-cell immunosuppression by inducing apoptosis-associated secretion of IL-2 in FLT3-ITD-mutated acute myeloid leukemia
Source: Transl Oncol. 2026 Jul 20;71:102894. doi: 10.1016/j.tranon.2026.102894 (PMC13392556; doi:10.1016/j.tranon.2026.102894)

Supplementary Figure 1.

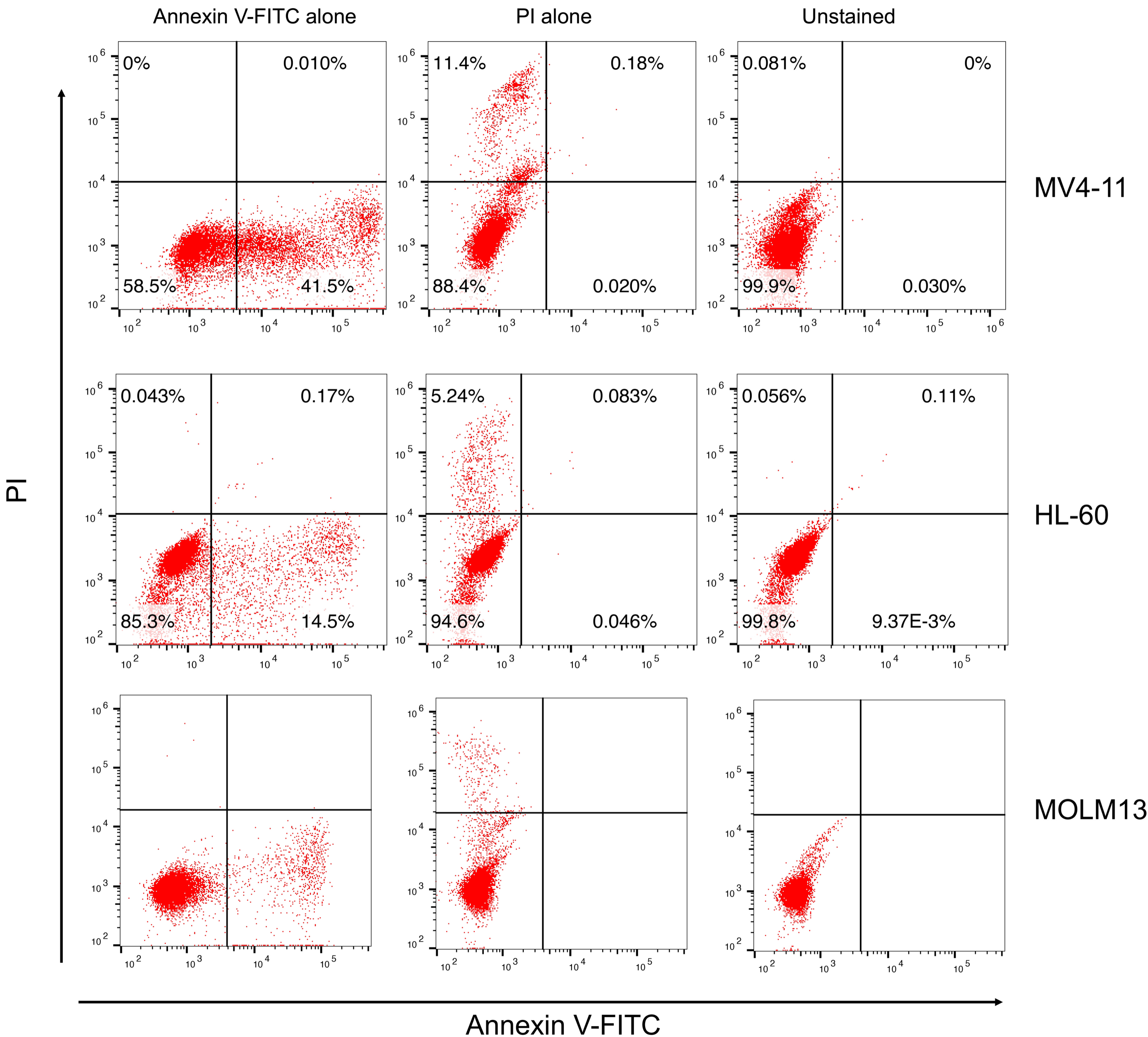

Supplement: Supplementary file 1 — Supplementary Figure 1. Compensation controls for Annexin V/PI staining in MV4–11, HL-60, and MOLM13 cells. [file mmc1.pdf]

Supplementary Figure 2.

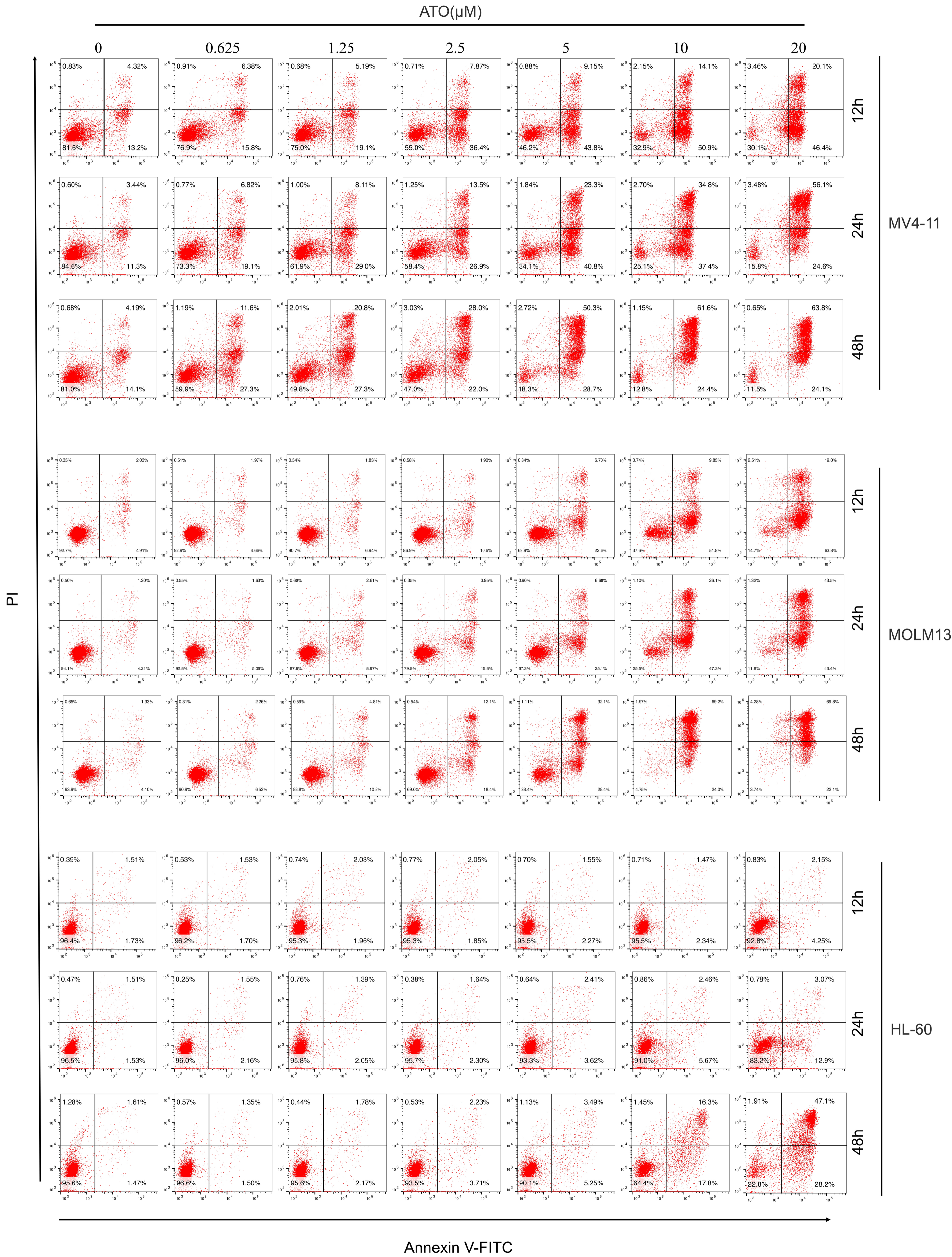

Supplement: Supplementary file 2 — Supplementary Figure 2. The apoptotic effects of ATO on MV4–11, MOLM13, and HL-60 cells. MV4–11, MOLM13, and HL-60 cells were exposed to different concentrations of ATO (0, 0.625, 1.25, 2.5, 5, 10, 20 µM) for 12, 24, and 48 h, and apoptosis was detected using Annexin V-FITC/PI staining. [file mmc2.pdf]

Supplementary Figure 3.

MV4-11

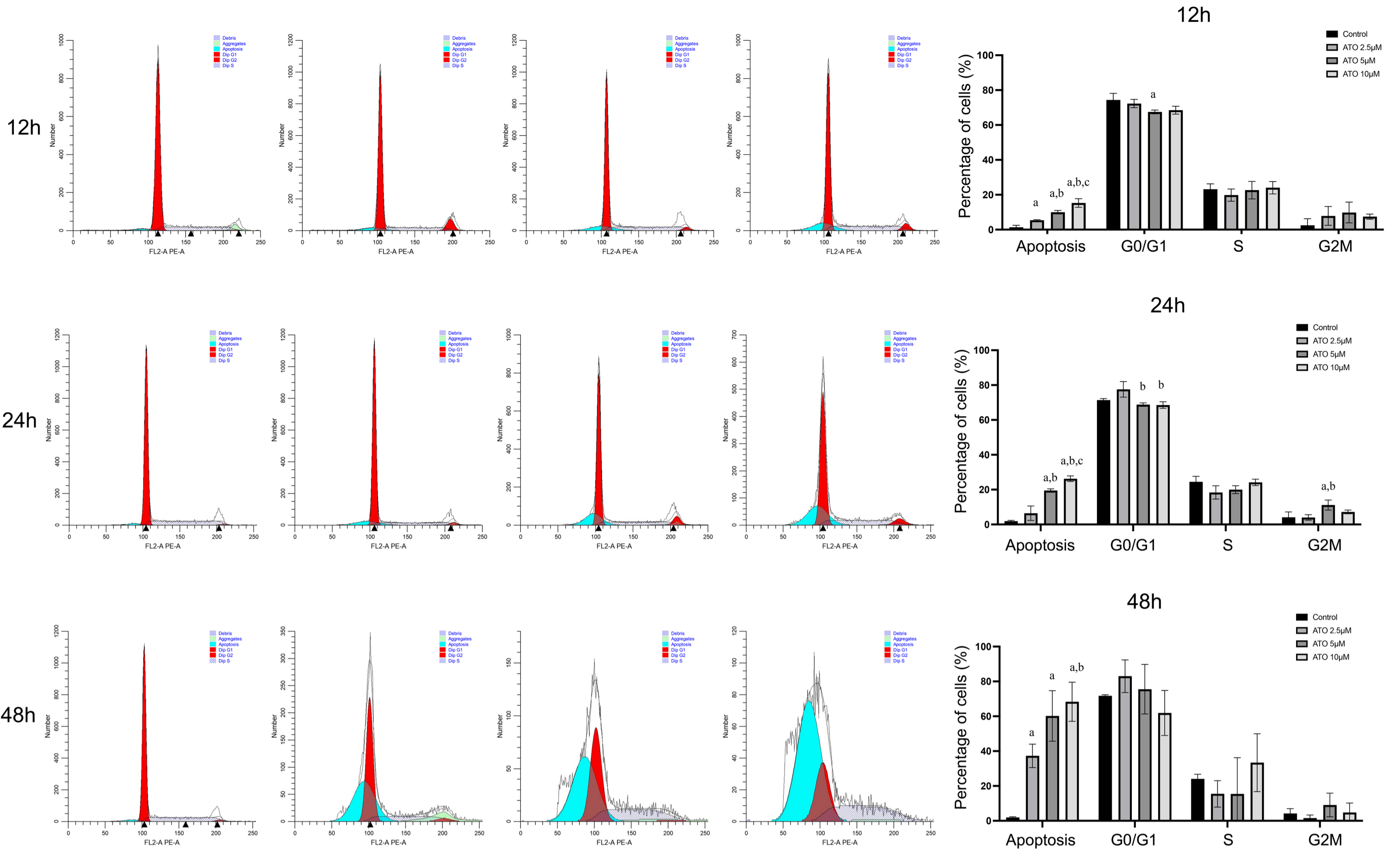

MOLM13

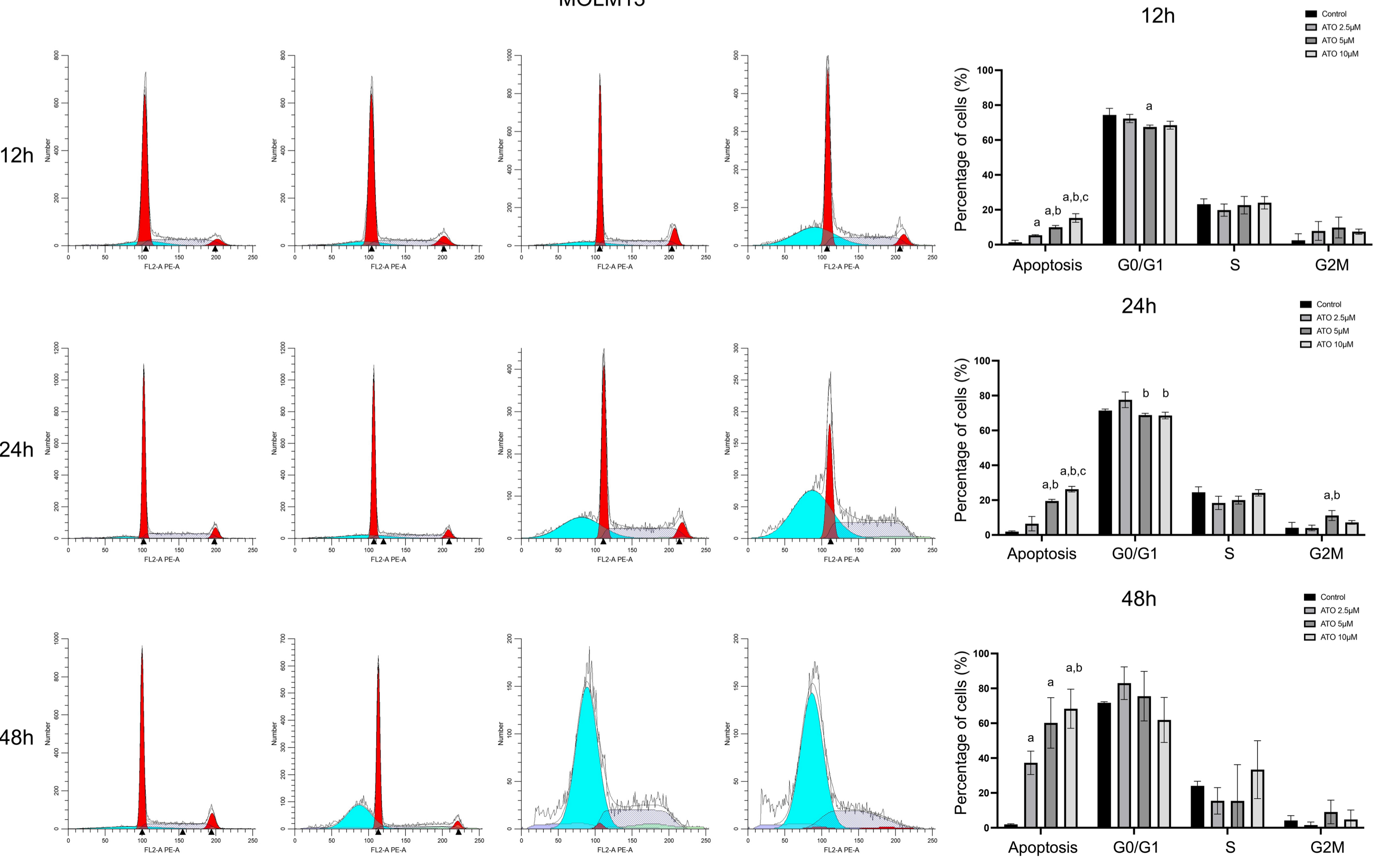

Supplement: Supplementary file 3 — Supplementary Figure 3. The effect of ATO on the cell cycle of FLT3-ITD-positive cells. The effects of ATO on the Apoptosis, G0/G1, S and G2/M phases of MV4–11 and MOLM13 cells following treatment with different concentrations (0, 2.5, 5, 10 μM) for 12, 24 and 48 h. a: significant versus control, b: significant versus ATO 2.5 μM, c: significant versus ATO 5 μM. [file mmc3.pdf]

Supplementary Figure 4.

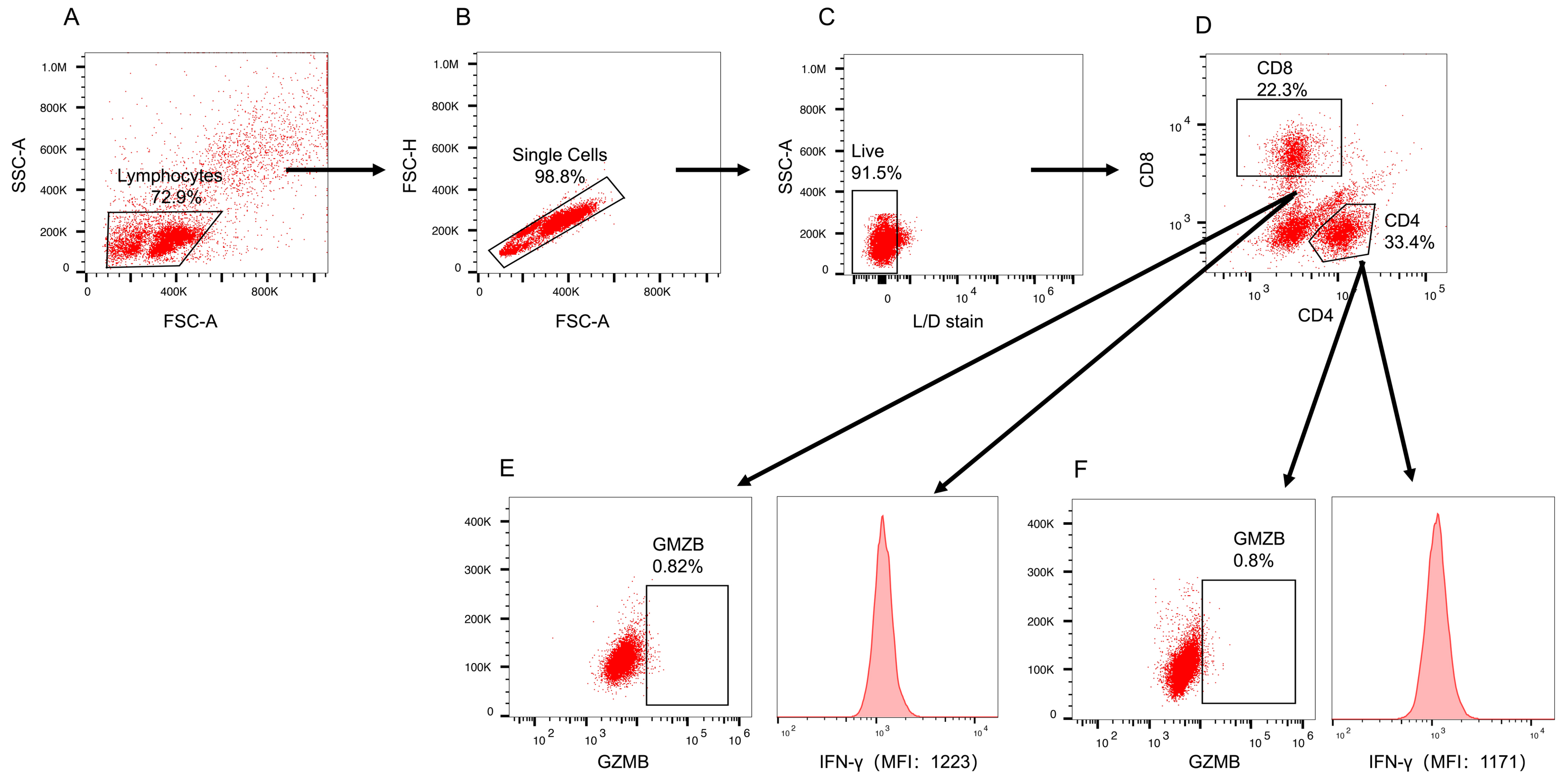

Supplement: Supplementary file 4 — Supplementary Figure 4. Typical gating strategies for CD4⁺/CD8⁺ T cell analysis. (A) Exclusion of debris using FSC‑A/SSC‑A; (B) Identification of single cells using FSC‑A/FSC‑H; (C) Selection of live cells using live/dead staining; (D) Definition of CD4⁺ and CD8⁺ T cell subsets using a CD4 versus CD8 plot; (E) Measurement of GZMB and IFN-γ levels in CD8⁺ T cells; (F) Measurement of GZMB and IFN-γ levels in CD4⁺ T cells. [file mmc4.pdf]

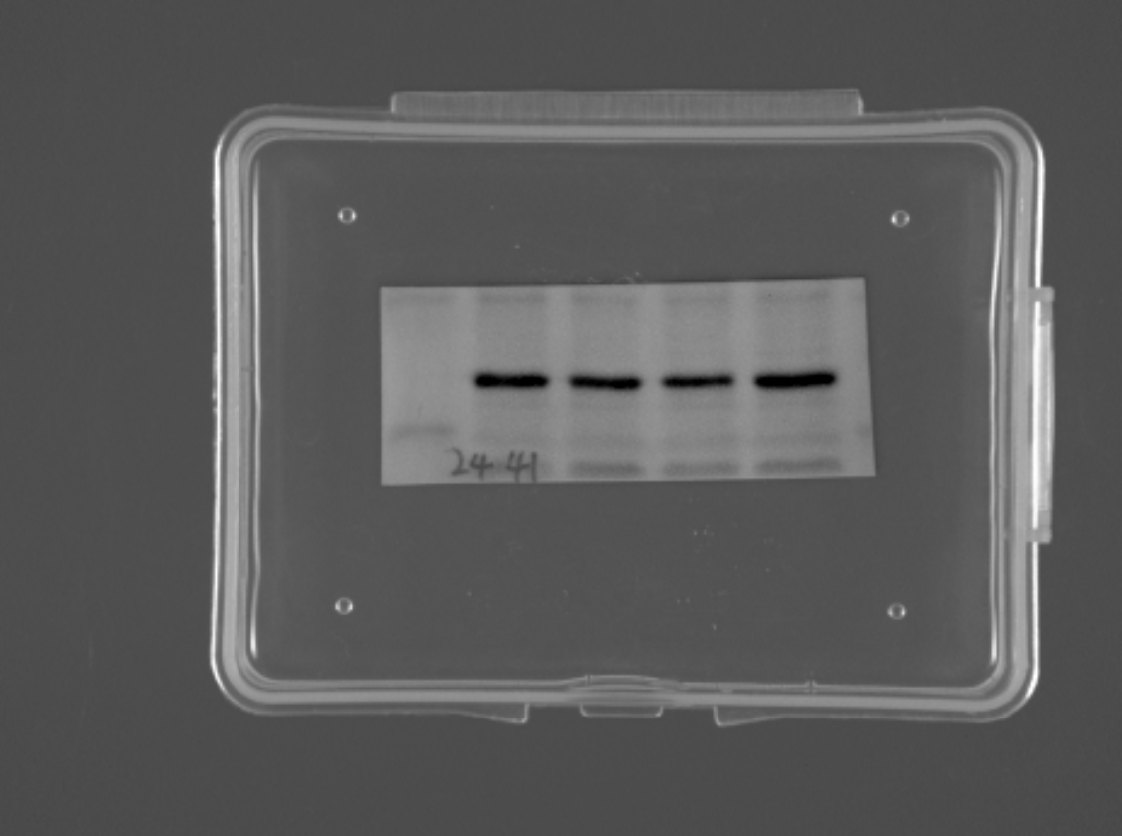

Supplement: Supplementary file 5 [file mmc5.zip › Original Images of Western blot/Fig.1/Fig.1A Bax.tif]

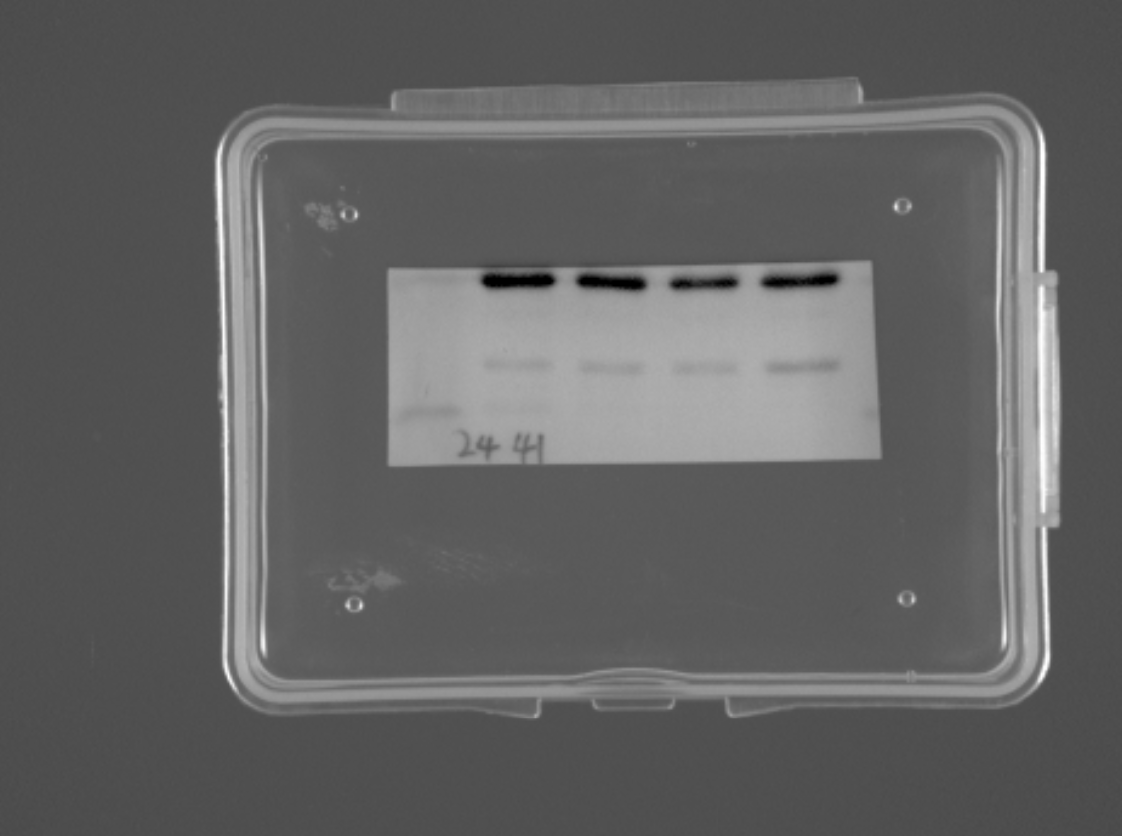

Supplement: Supplementary file 5 [file mmc5.zip › Original Images of Western blot/Fig.1/Fig.1A Bcl2.tif]

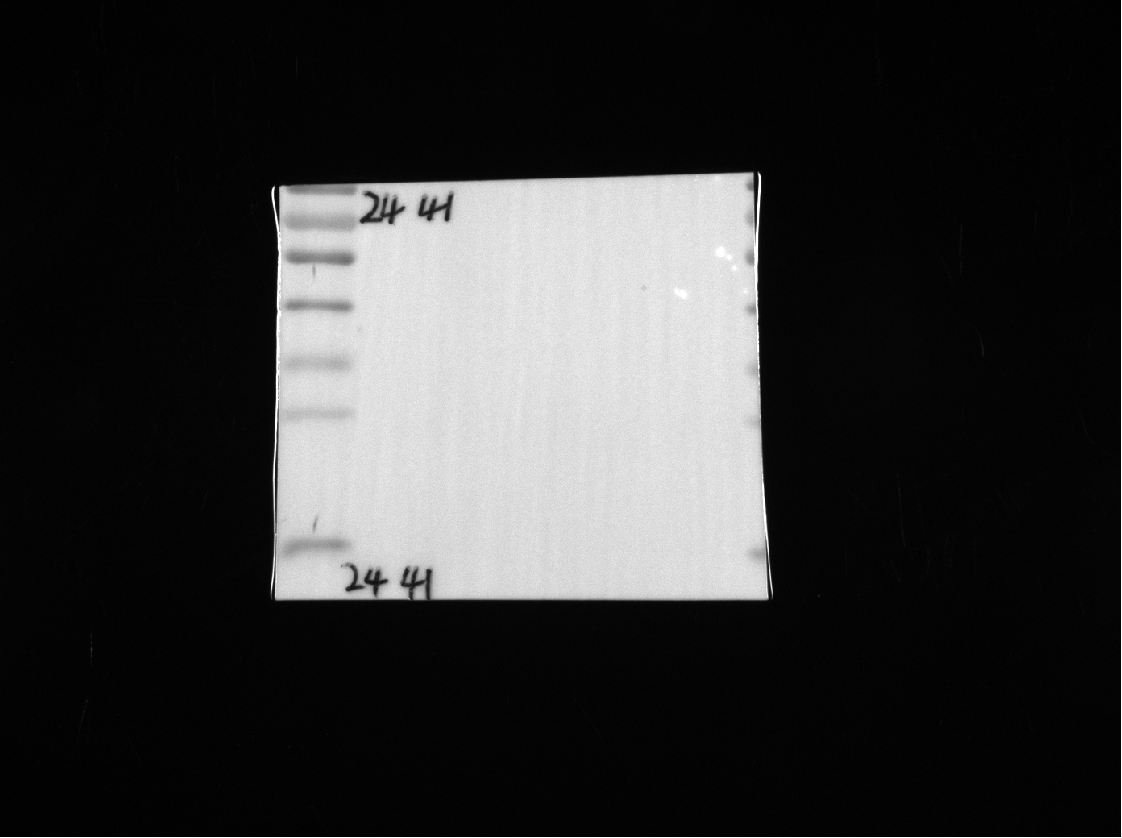

Supplement: Supplementary file 5 [file mmc5.zip › Original Images of Western blot/Fig.1/Fig.1A Full membrane.tif]

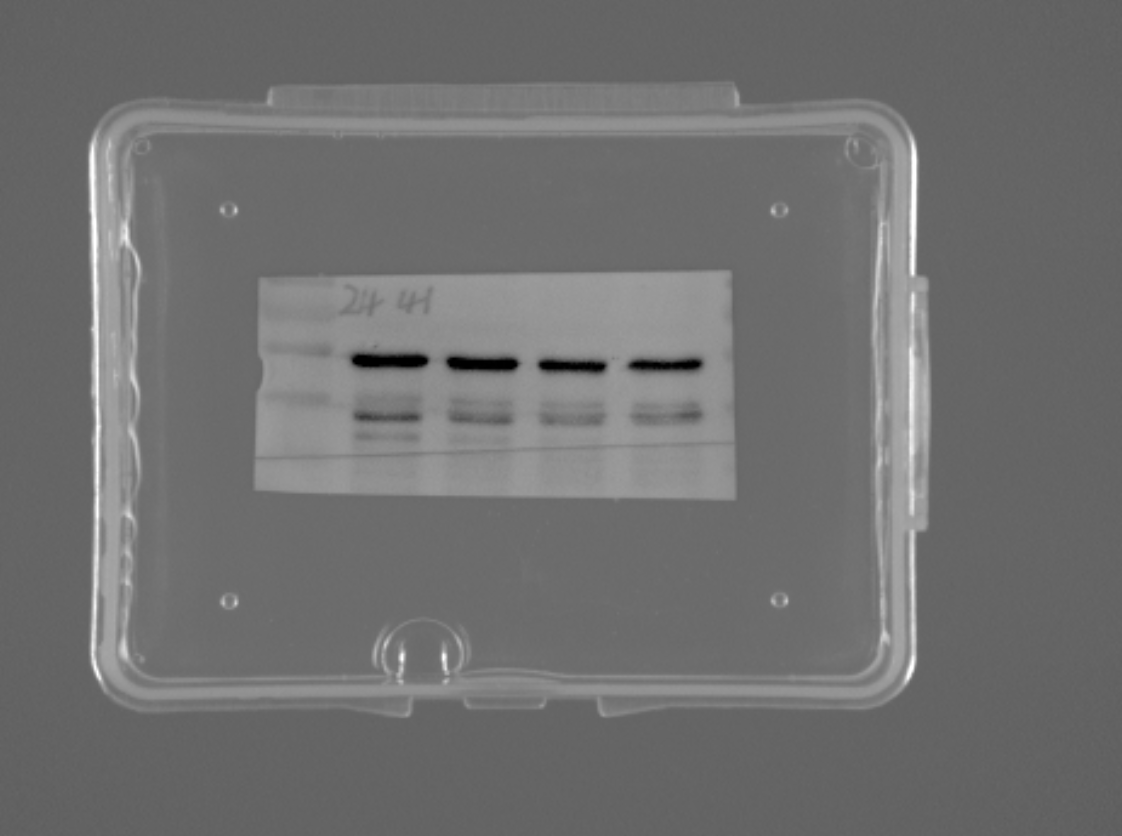

Supplement: Supplementary file 5 [file mmc5.zip › Original Images of Western blot/Fig.1/Fig.1A Tubulin.scn.tif]

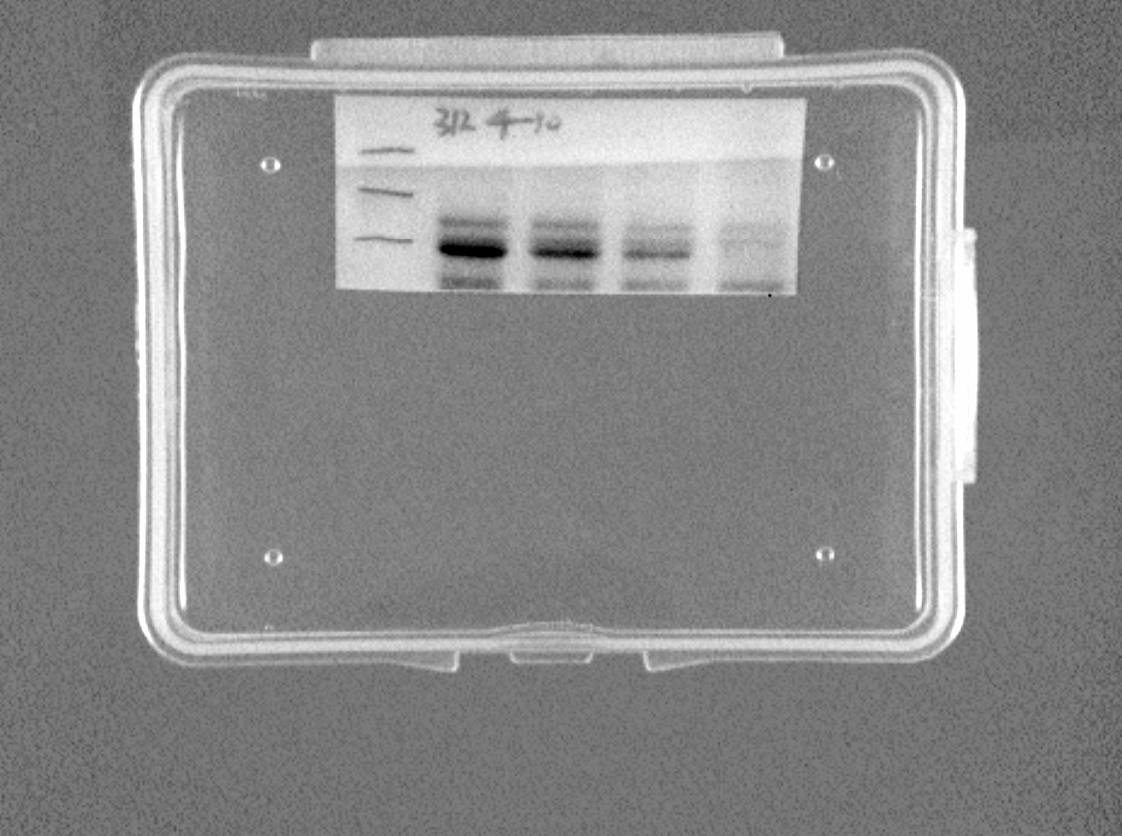

Supplement: Supplementary file 5 [file mmc5.zip › Original Images of Western blot/Fig.1/Fig.1B Caspase3.tif]

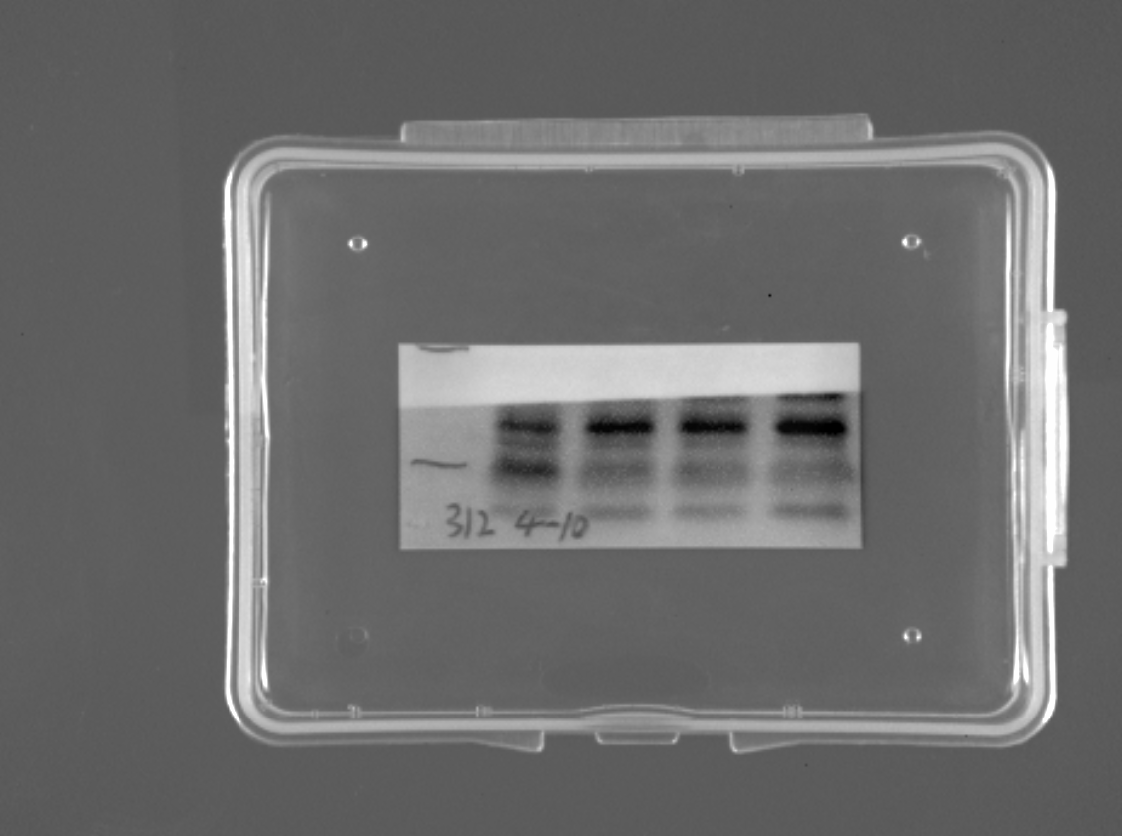

Supplement: Supplementary file 5 [file mmc5.zip › Original Images of Western blot/Fig.1/Fig.1B Cleaved-caspase3.tif]

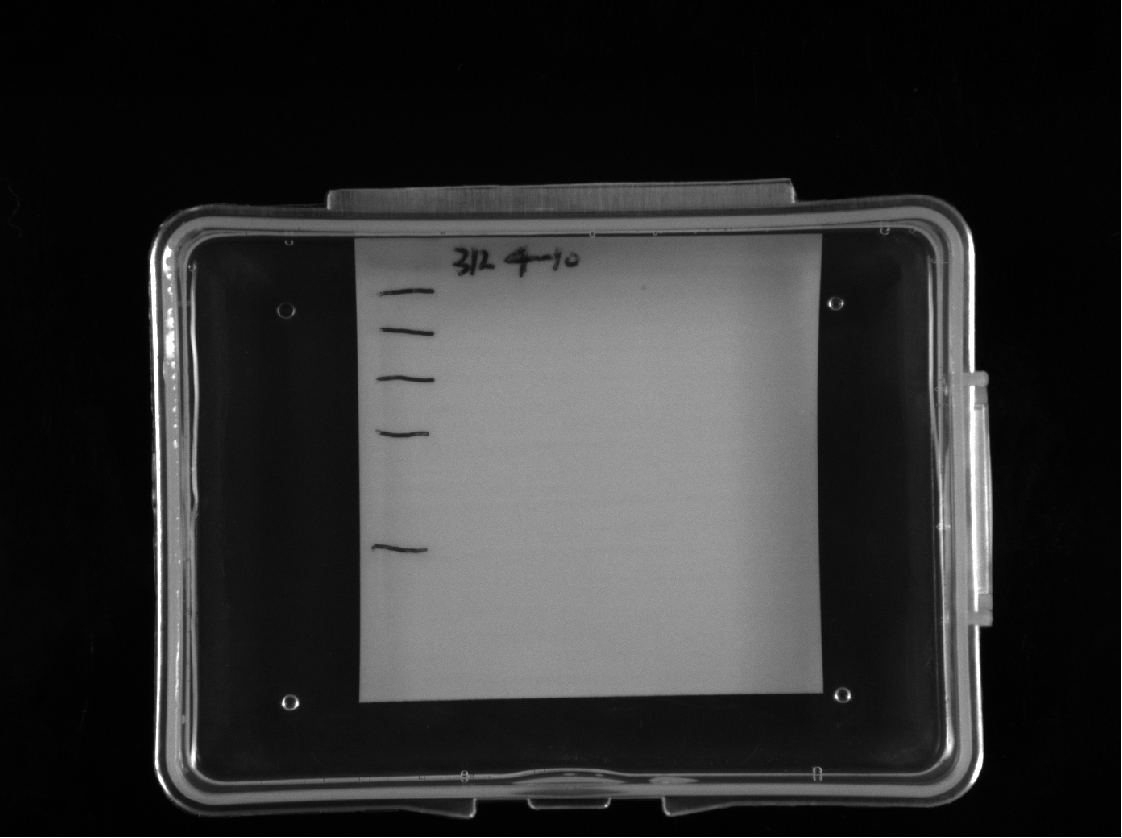

Supplement: Supplementary file 5 [file mmc5.zip › Original Images of Western blot/Fig.1/Fig.1B Full membrane.tif]

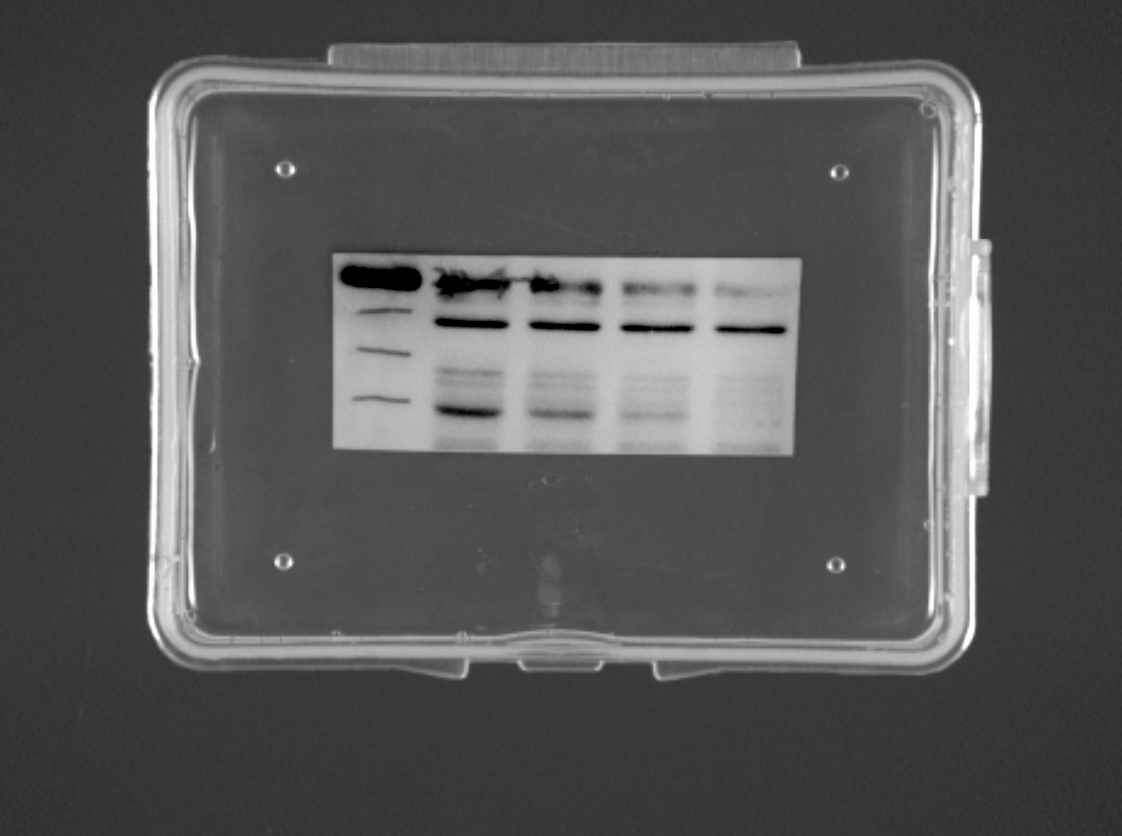

Supplement: Supplementary file 5 [file mmc5.zip › Original Images of Western blot/Fig.1/Fig.1B Tubulin.tif]

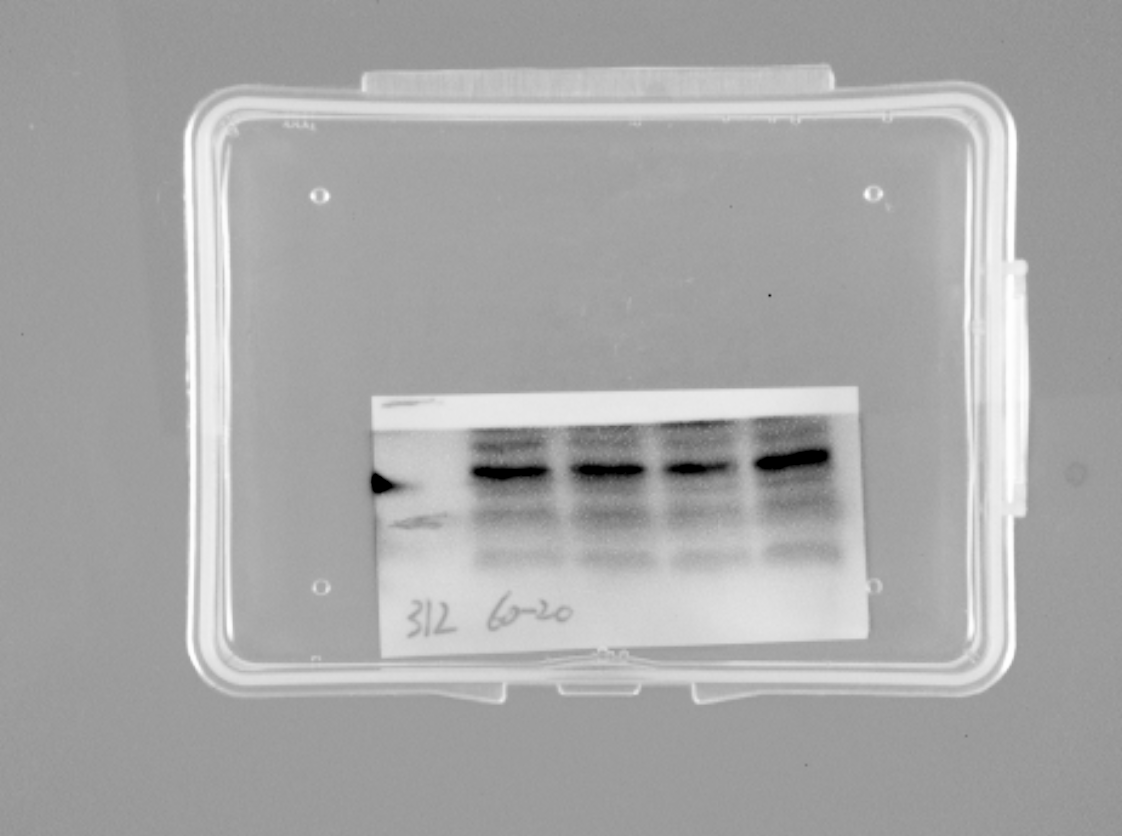

Supplement: Supplementary file 5 [file mmc5.zip › Original Images of Western blot/Fig.1/Fig.1C Bax.tif]

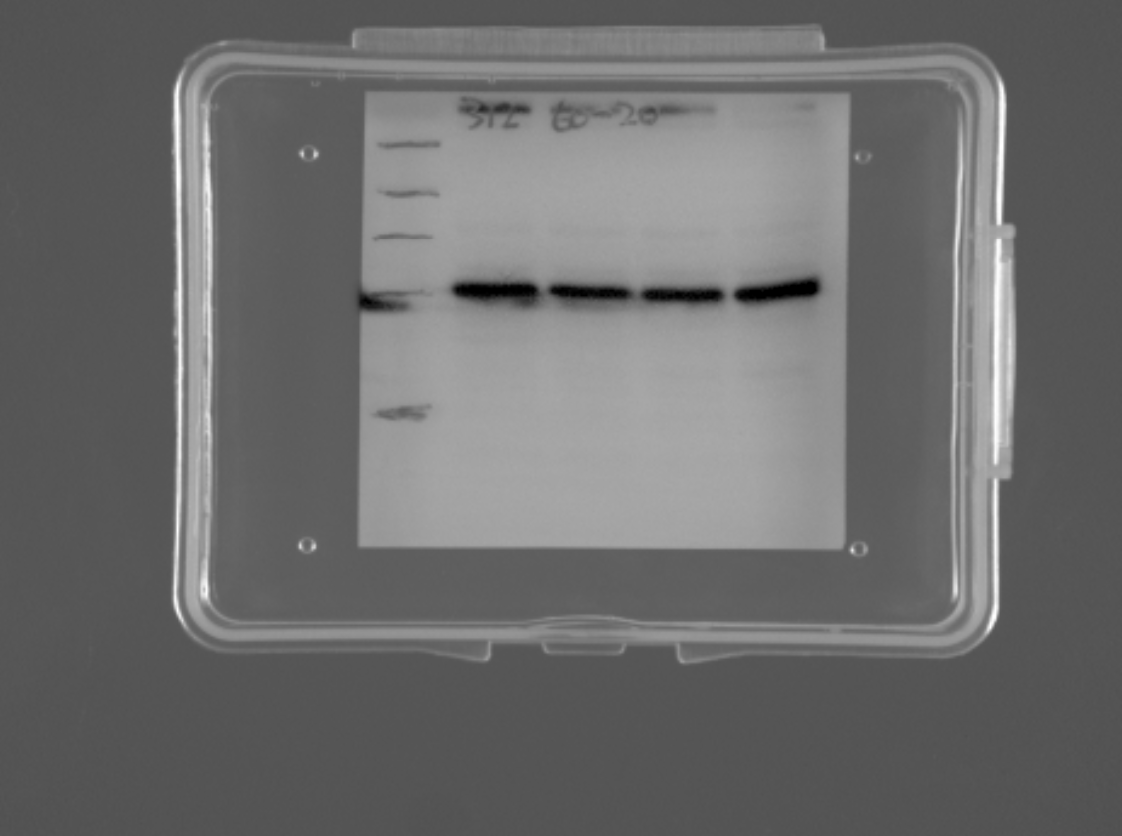

Supplement: Supplementary file 5 [file mmc5.zip › Original Images of Western blot/Fig.1/Fig.1C Bcl2.tif]

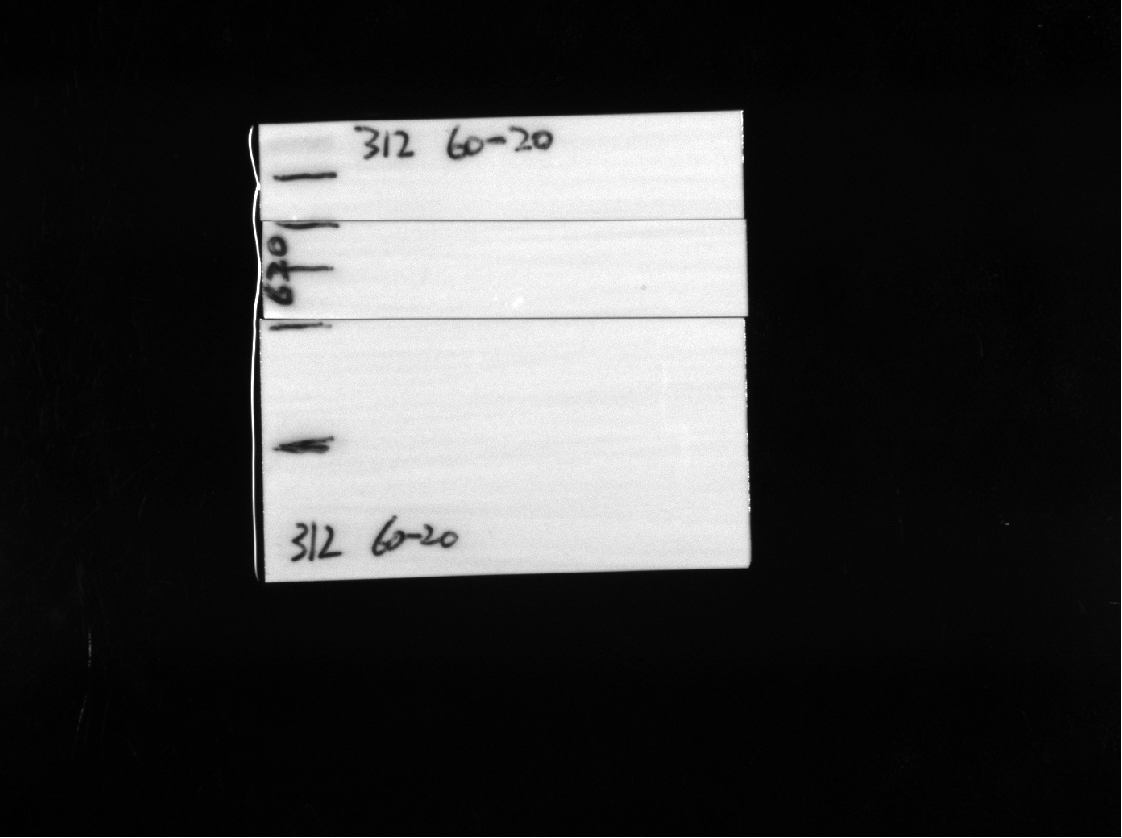

Supplement: Supplementary file 5 [file mmc5.zip › Original Images of Western blot/Fig.1/Fig.1C Full membrane.tif]

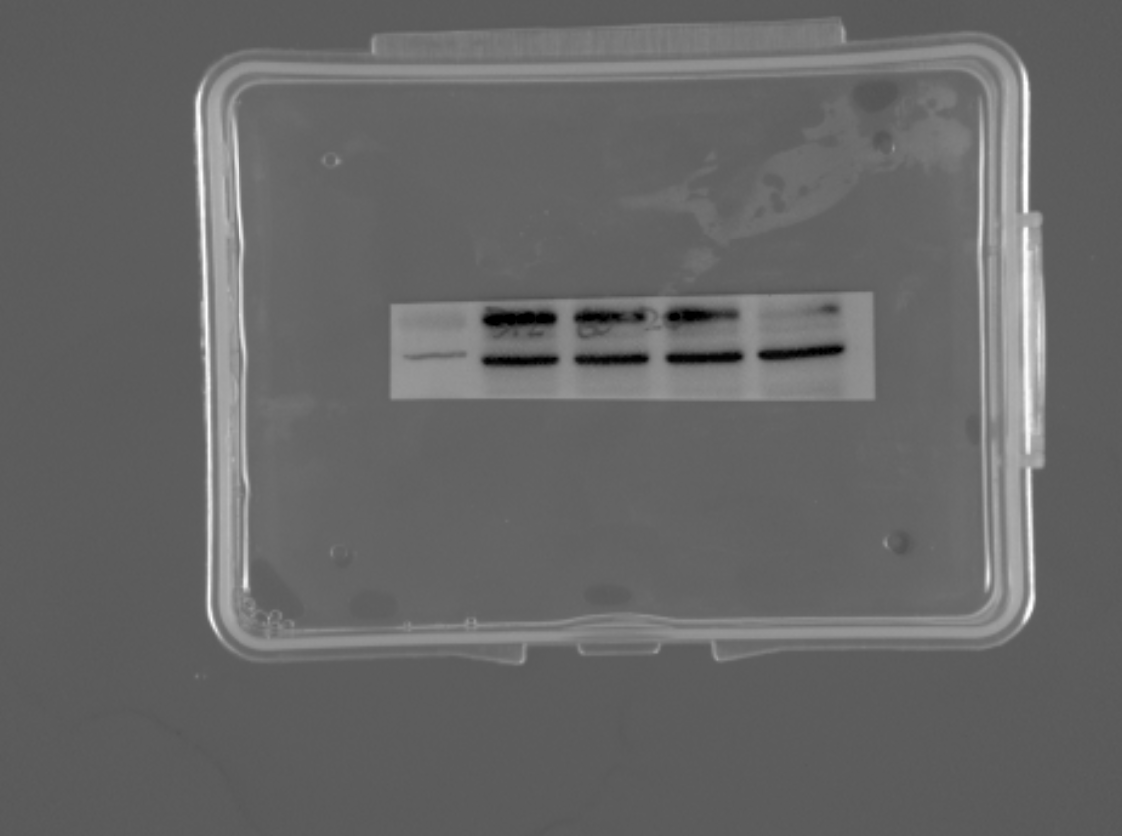

Supplement: Supplementary file 5 [file mmc5.zip › Original Images of Western blot/Fig.1/Fig.1C Tubulin.tif]

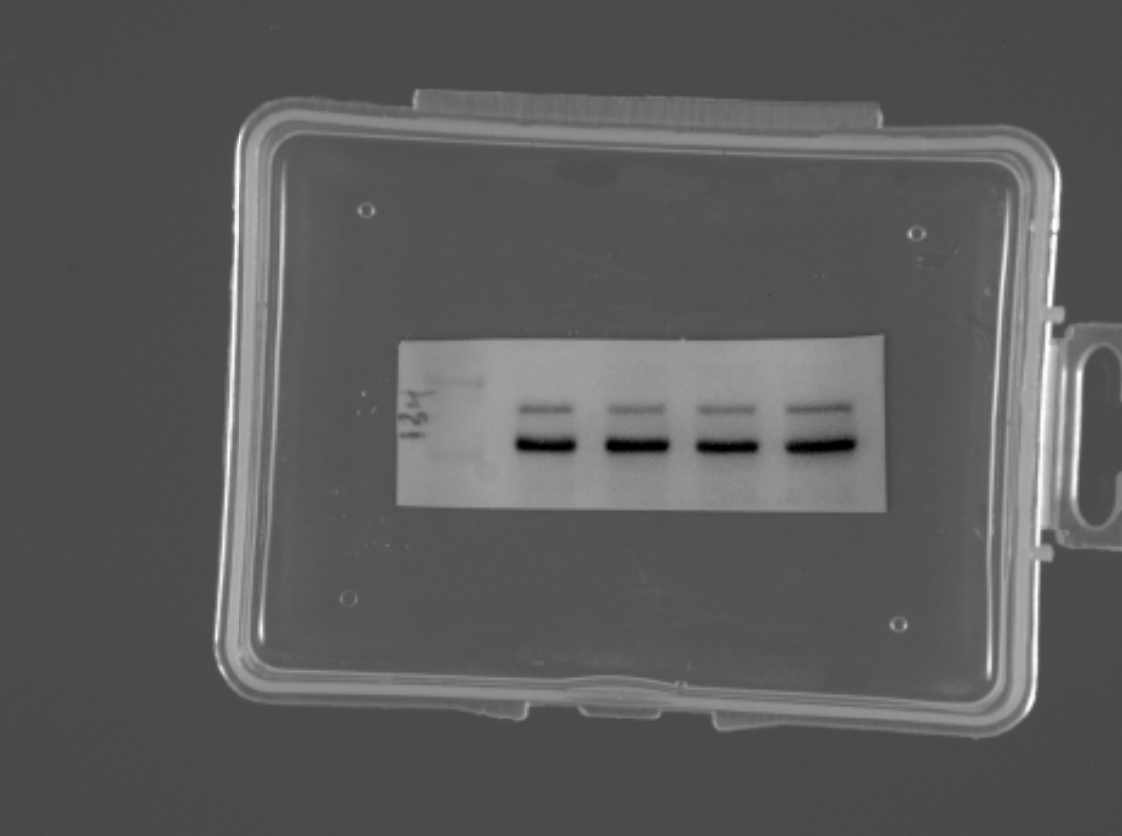

Supplement: Supplementary file 5 [file mmc5.zip › Original Images of Western blot/Fig.1/Fig.1D Caspas3.tif]

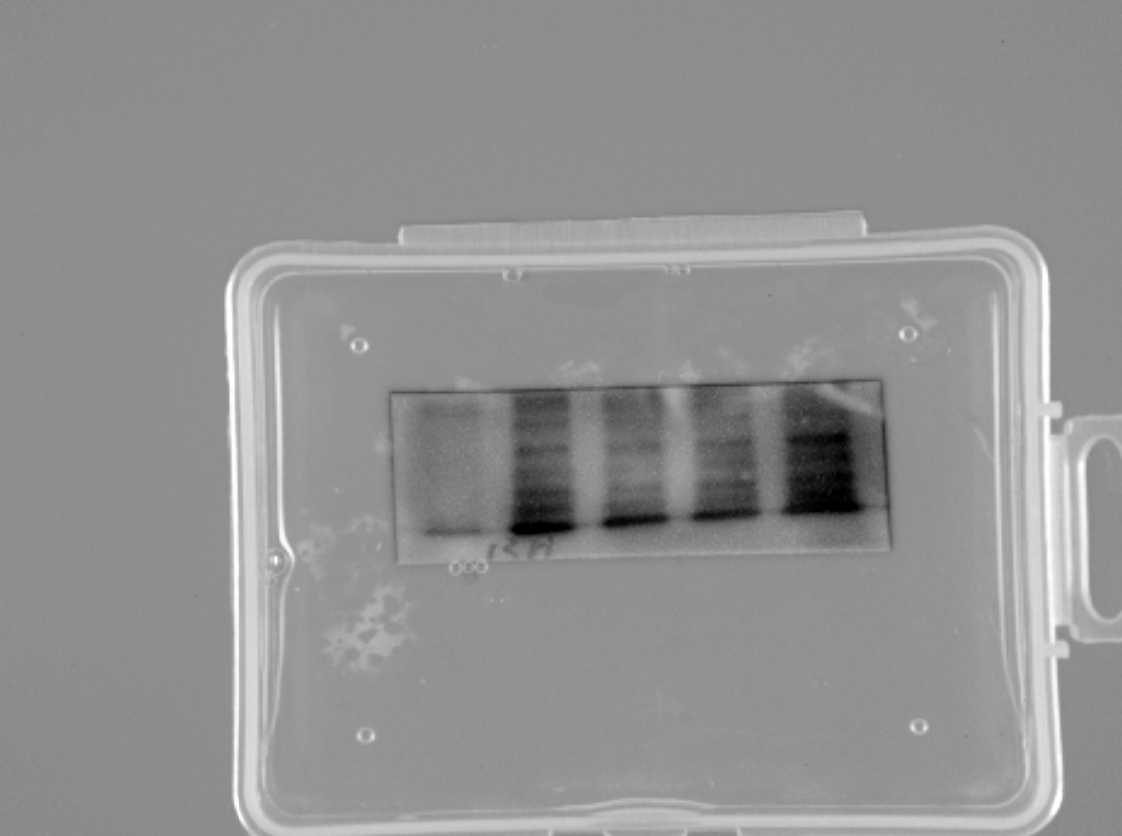

Supplement: Supplementary file 5 [file mmc5.zip › Original Images of Western blot/Fig.1/Fig.1D Cleaved-caspase3.tif]

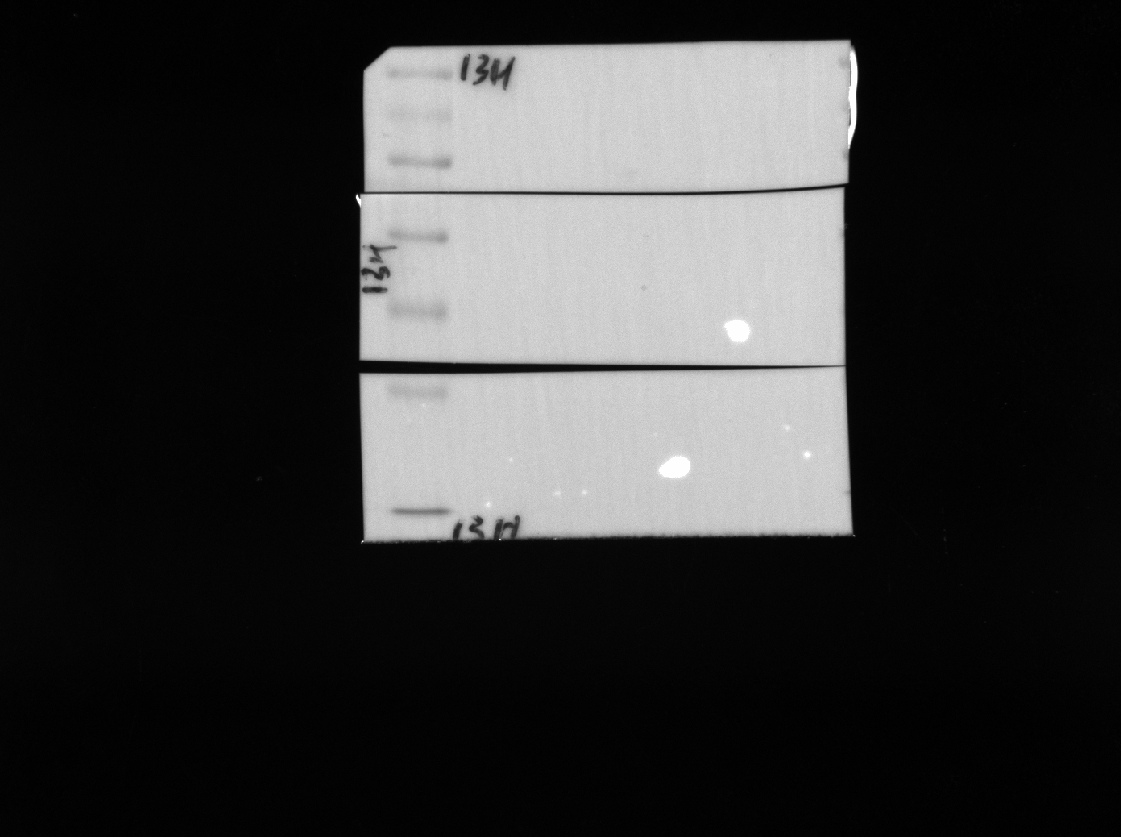

Supplement: Supplementary file 5 [file mmc5.zip › Original Images of Western blot/Fig.1/Fig.1D Full membrane.tif]

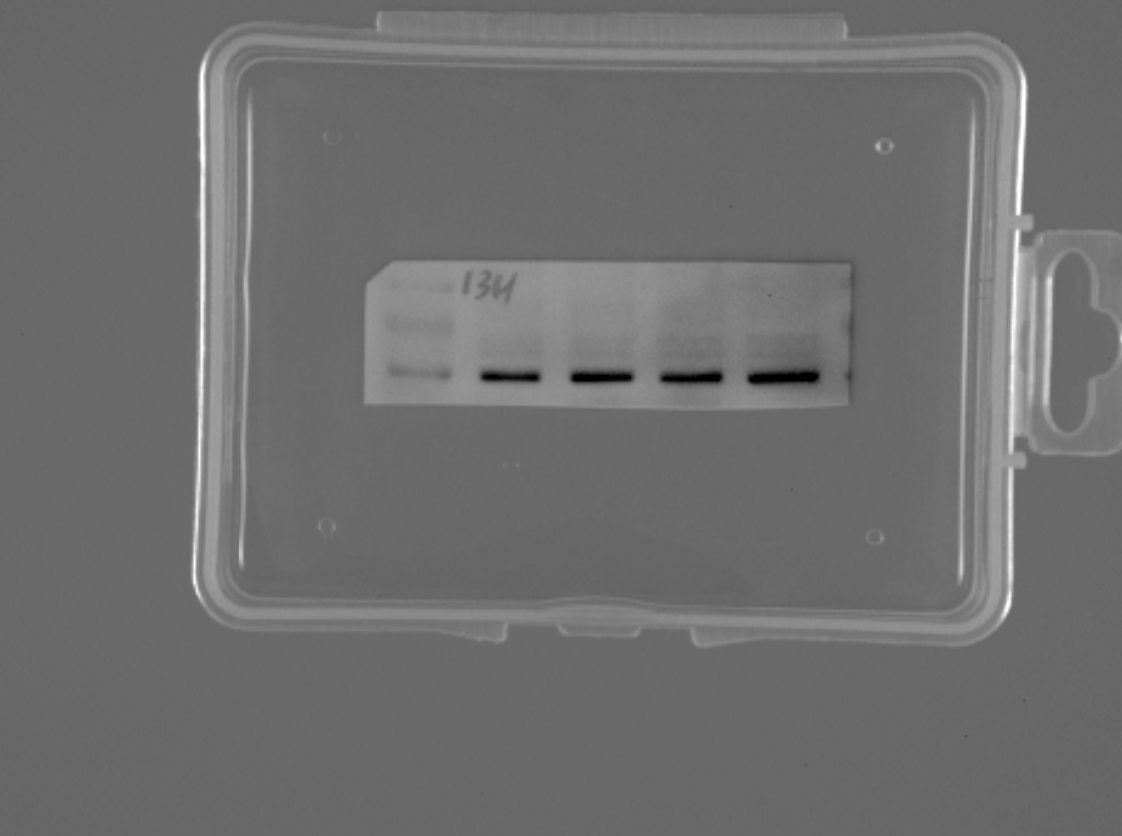

Supplement: Supplementary file 5 [file mmc5.zip › Original Images of Western blot/Fig.1/Fig.1D Tubulin.tif]

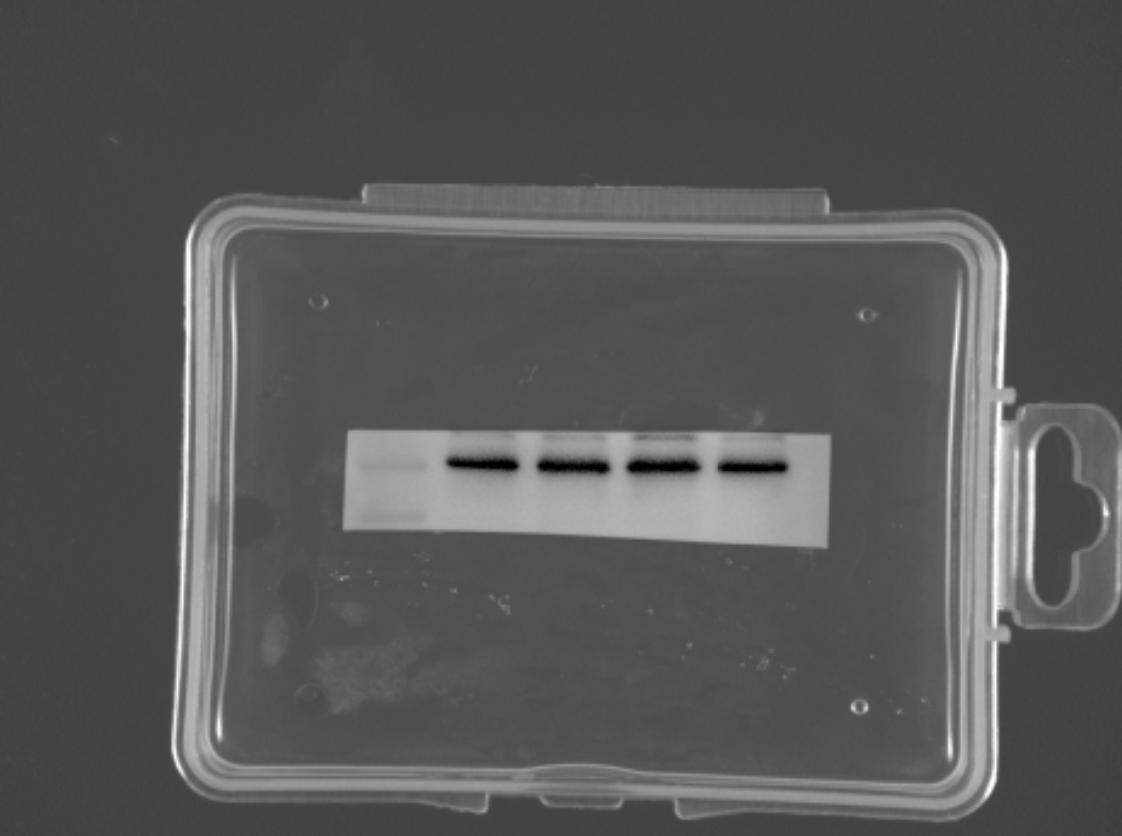

Supplement: Supplementary file 5 [file mmc5.zip › Original Images of Western blot/Fig.2/Fig.2 Caspase3.tif]

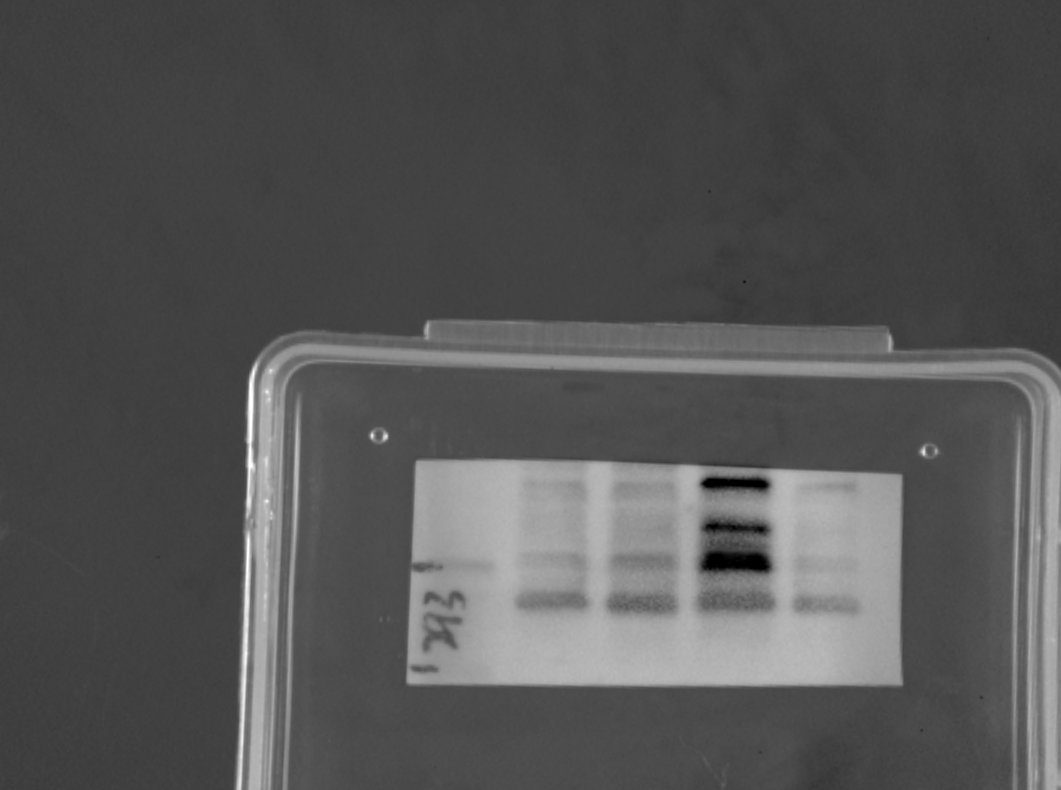

Supplement: Supplementary file 5 [file mmc5.zip › Original Images of Western blot/Fig.2/Fig.2 Cleaved-caspase3.tif]

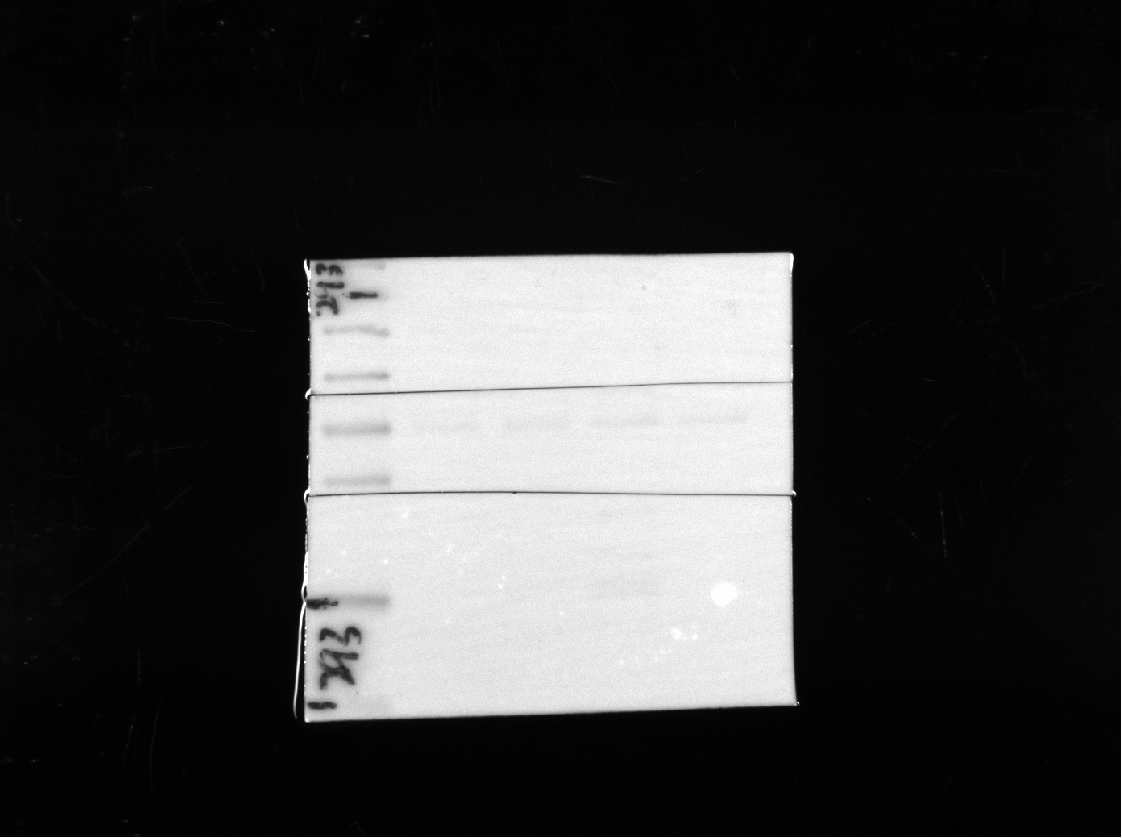

Supplement: Supplementary file 5 [file mmc5.zip › Original Images of Western blot/Fig.2/Fig.2 Full membrane.tif]

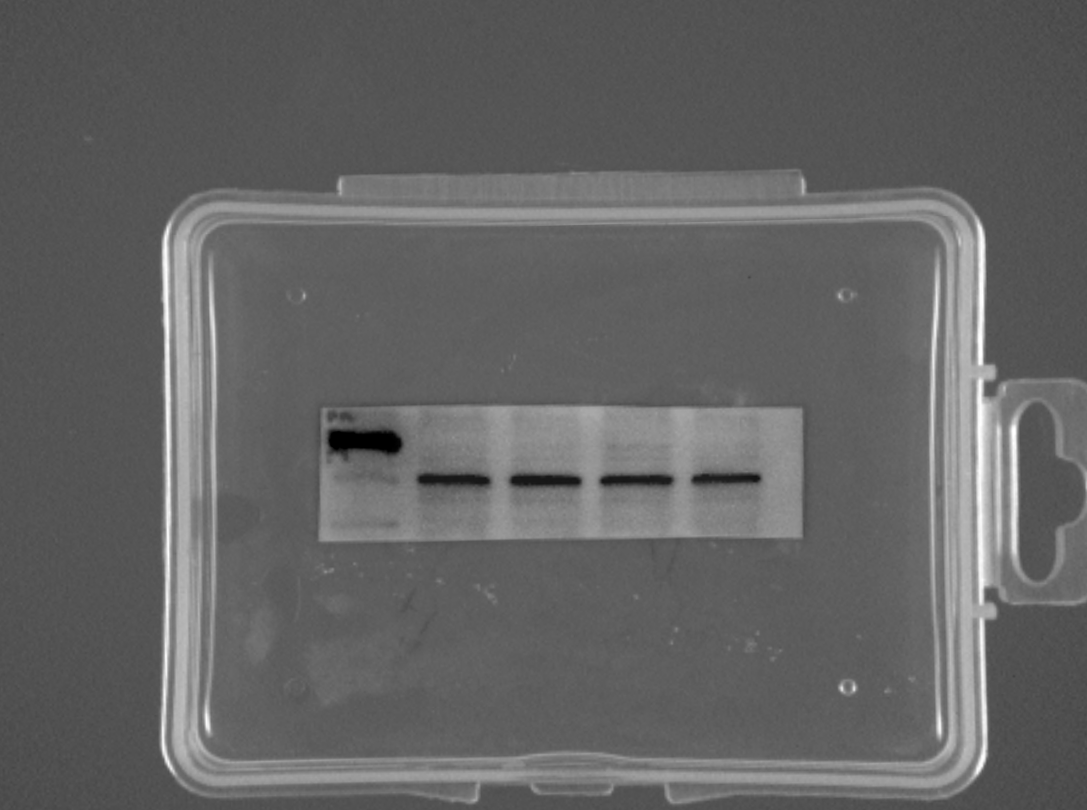

Supplement: Supplementary file 5 [file mmc5.zip › Original Images of Western blot/Fig.2/Fig.2 Tubulin.tif]

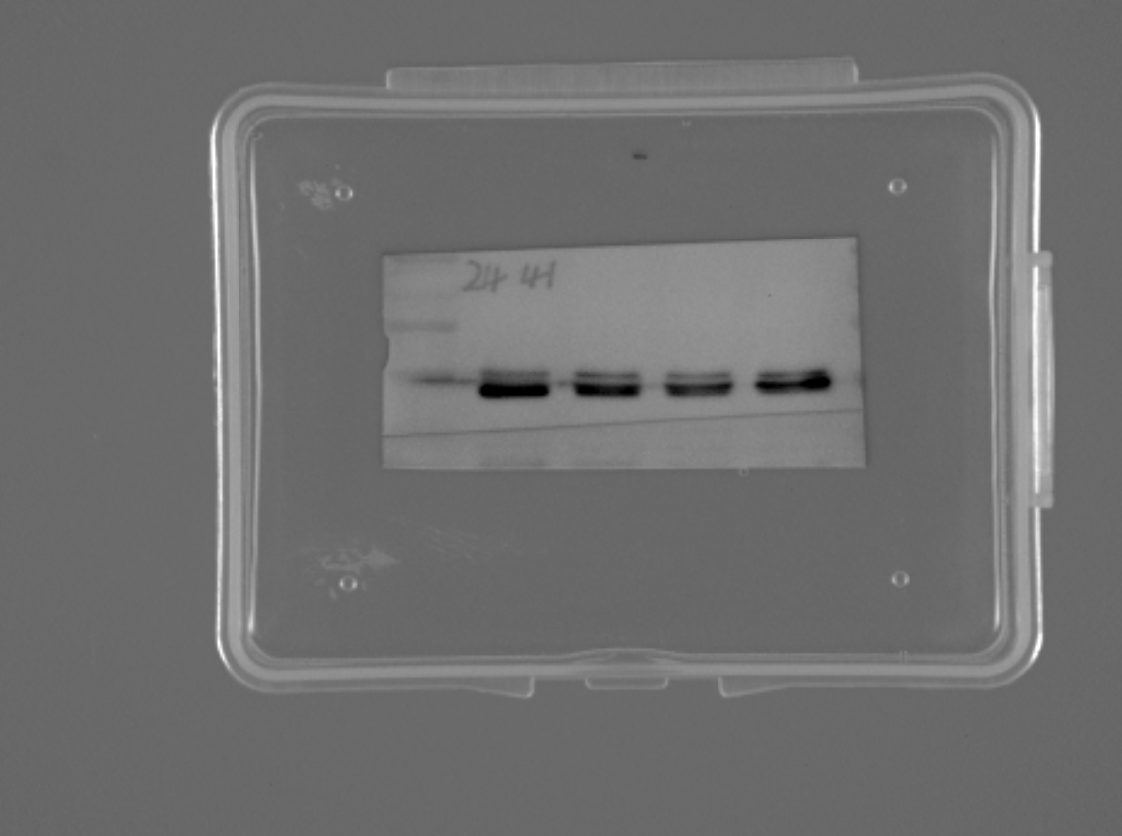

Supplement: Supplementary file 5 [file mmc5.zip › Original Images of Western blot/Fig.3/Fig.3A ERK.tif]

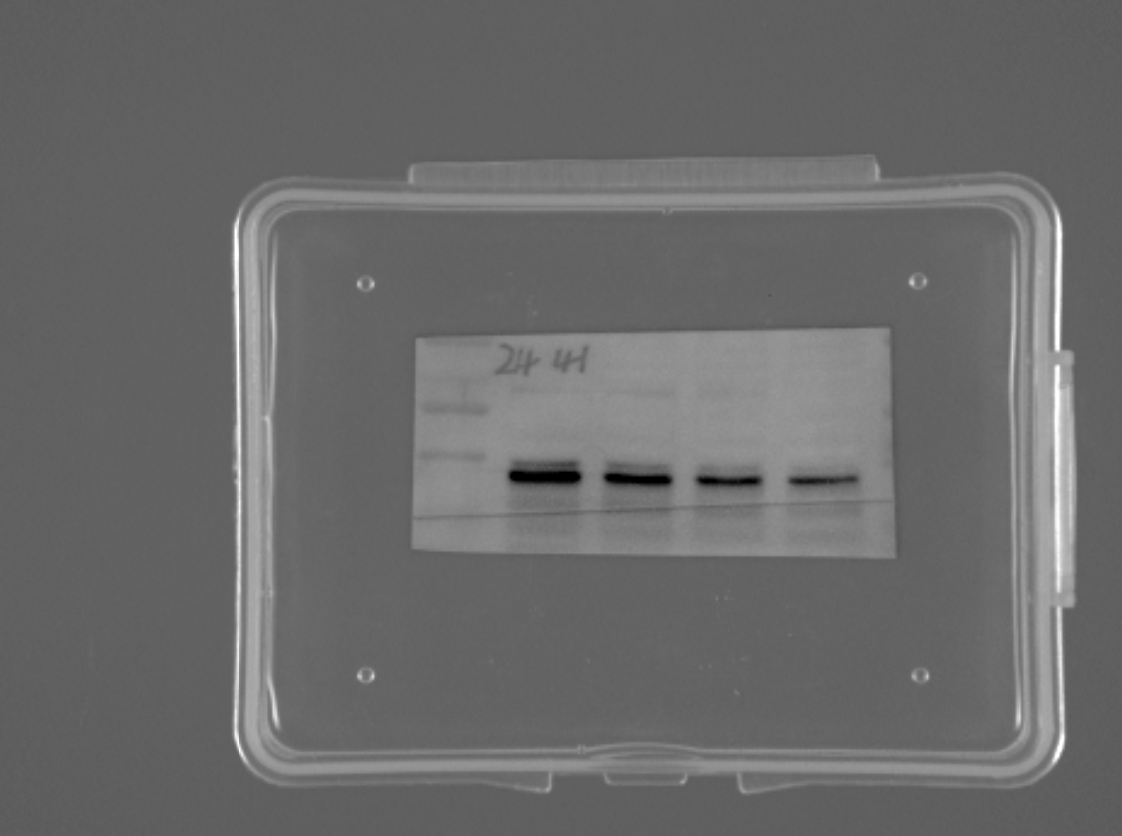

Supplement: Supplementary file 5 [file mmc5.zip › Original Images of Western blot/Fig.3/Fig.3A p-ERK.tif]

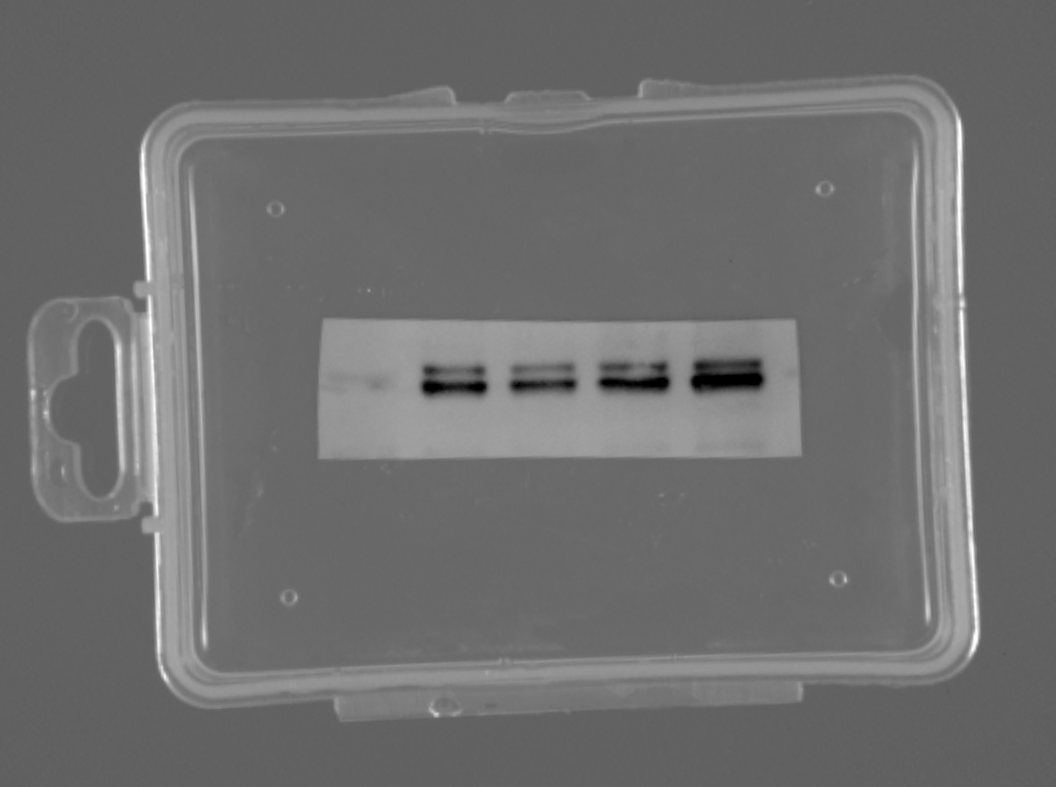

Supplement: Supplementary file 5 [file mmc5.zip › Original Images of Western blot/Fig.3/Fig.3B ERK.tif]

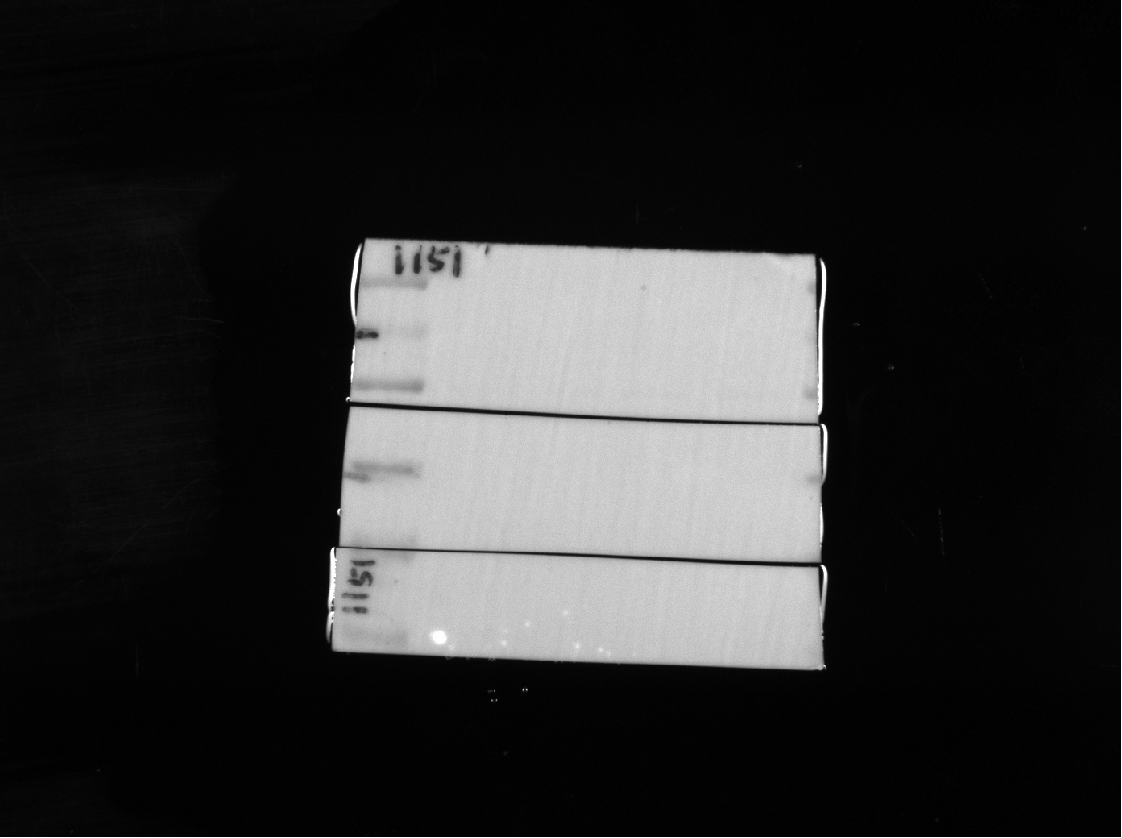

Supplement: Supplementary file 5 [file mmc5.zip › Original Images of Western blot/Fig.3/Fig.3B Full menbrane.tif]

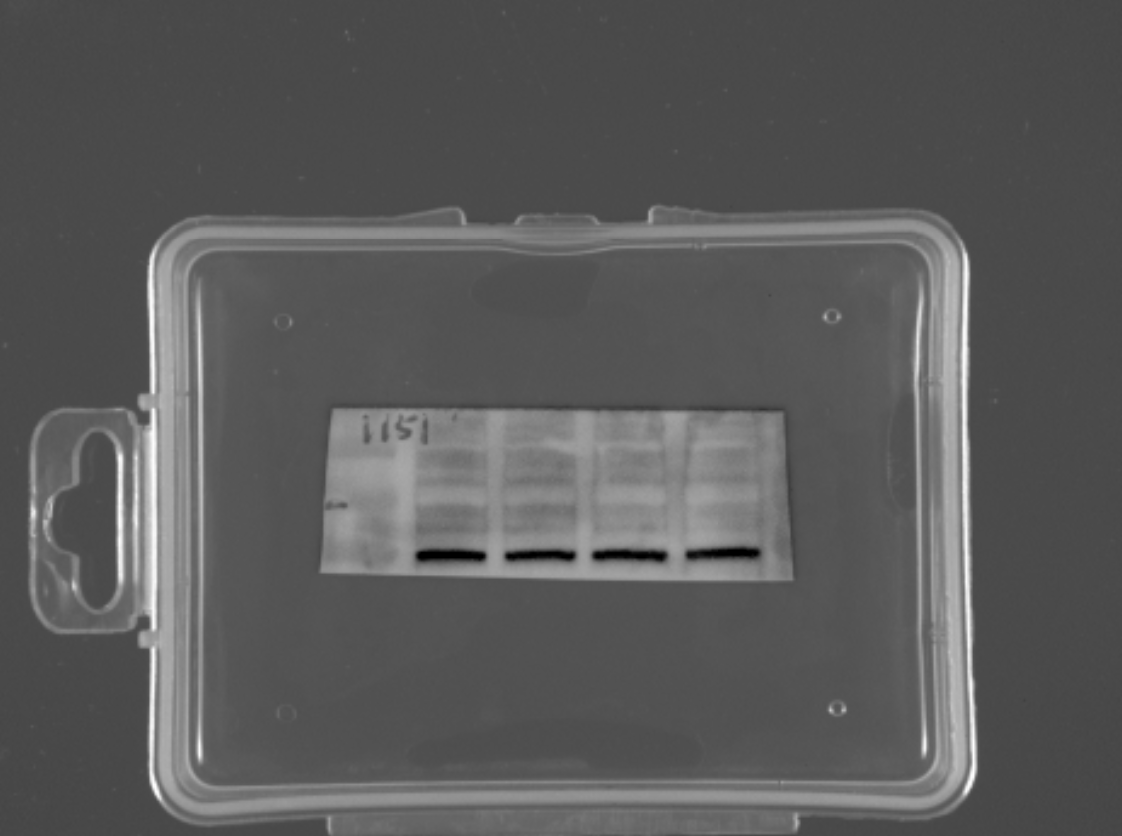

Supplement: Supplementary file 5 [file mmc5.zip › Original Images of Western blot/Fig.3/Fig.3B Tubulin.tif]

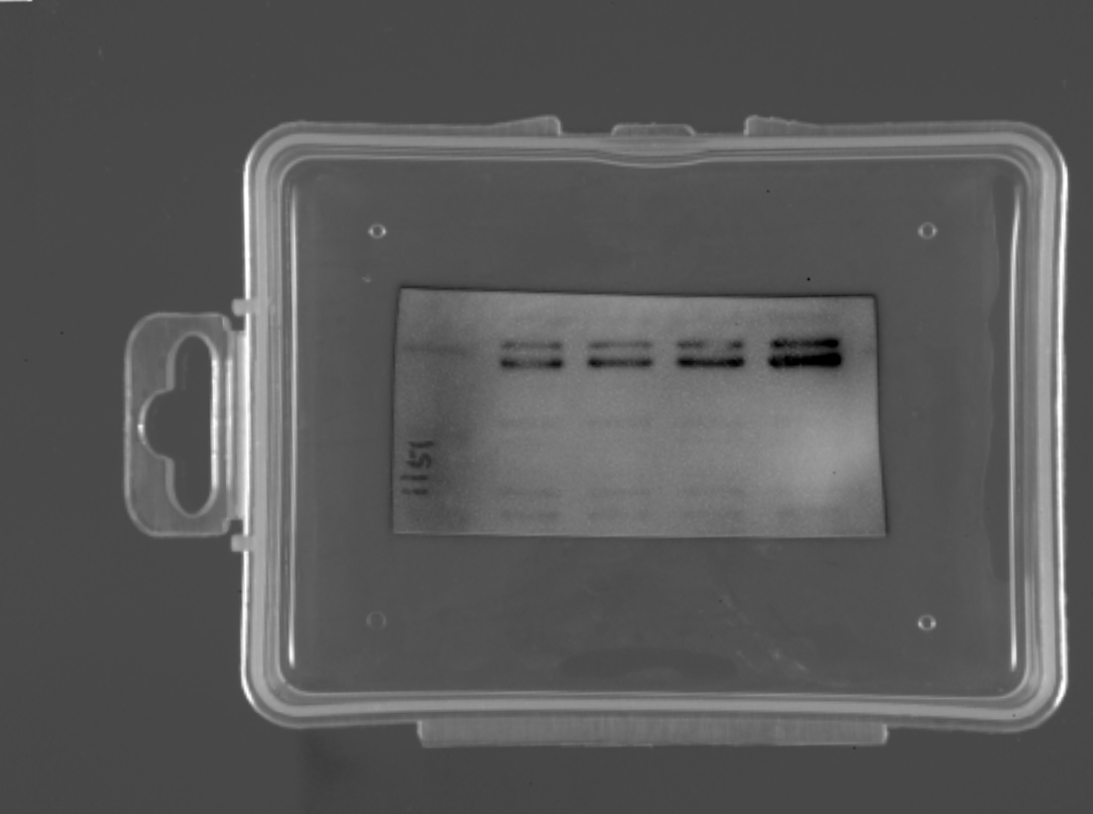

Supplement: Supplementary file 5 [file mmc5.zip › Original Images of Western blot/Fig.3/Fig.3B p-ERK.tif]

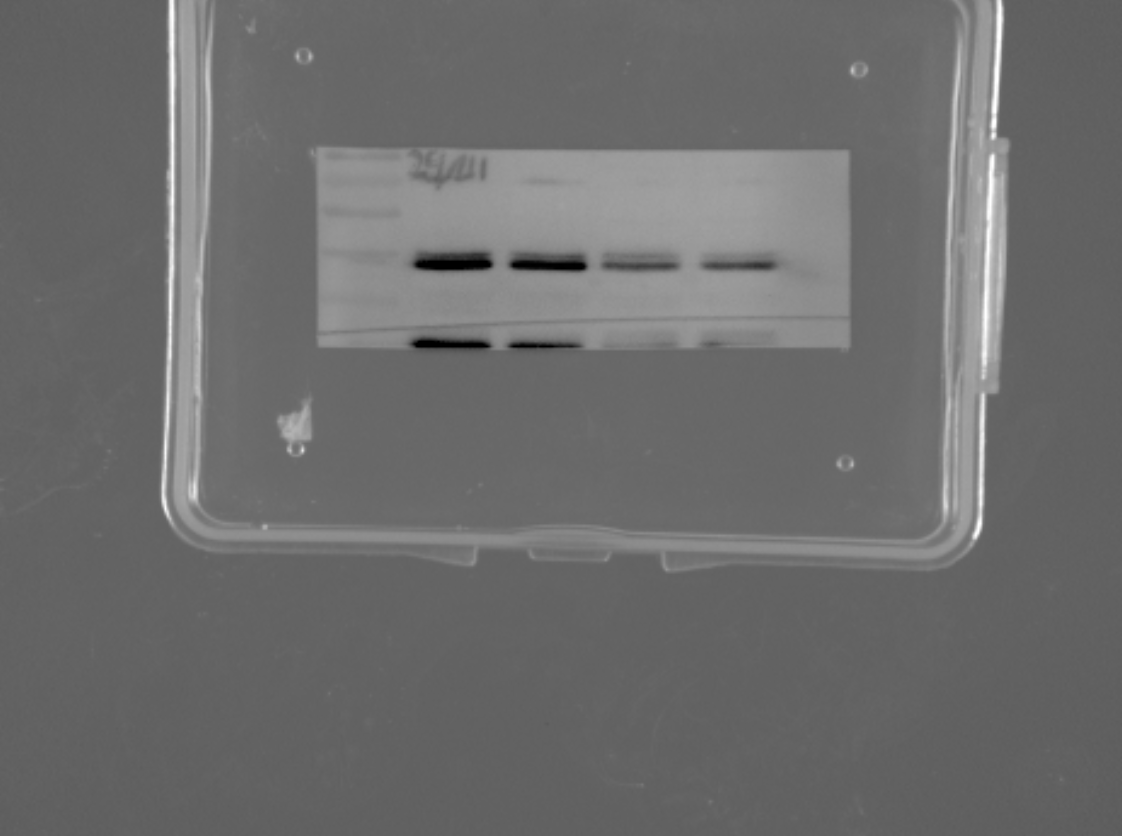

Supplement: Supplementary file 5 [file mmc5.zip › Original Images of Western blot/Fig.3/Fig.3C ERK.tif]

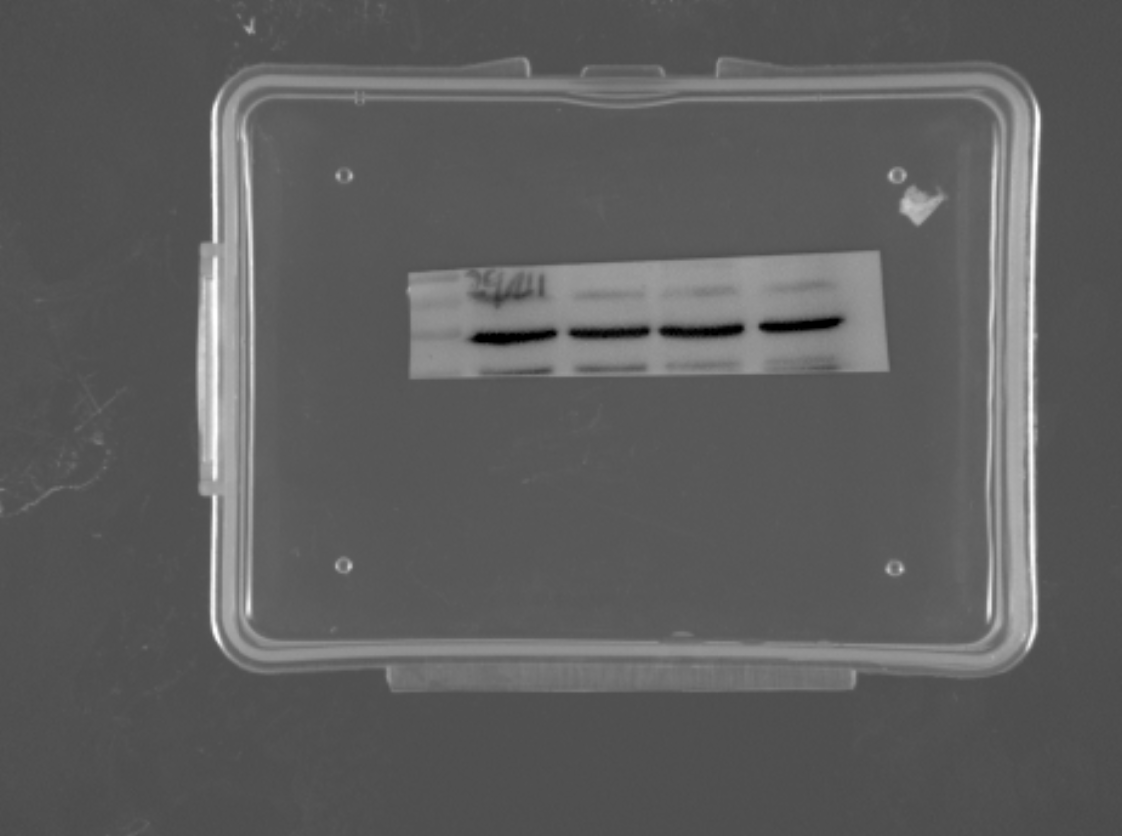

Supplement: Supplementary file 5 [file mmc5.zip › Original Images of Western blot/Fig.3/Fig.3C Tubulin.tif]

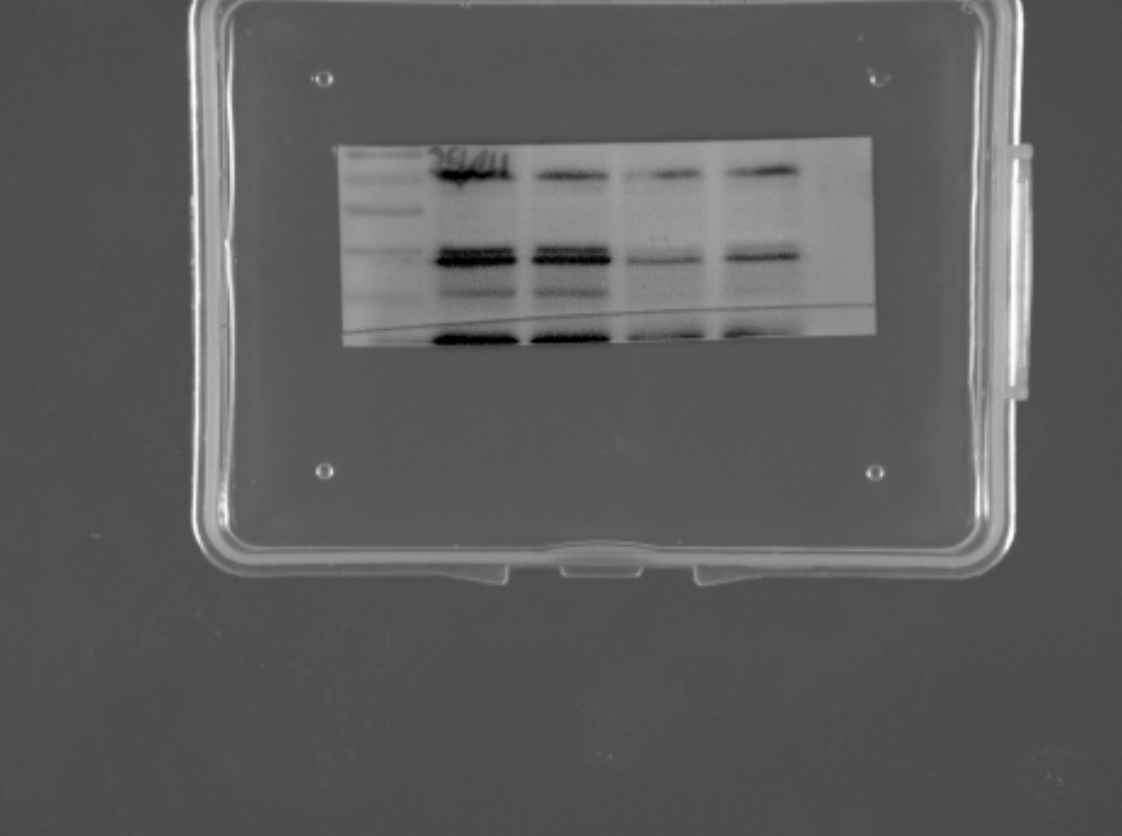

Supplement: Supplementary file 5 [file mmc5.zip › Original Images of Western blot/Fig.3/Fig.3C p-ERK.tif]

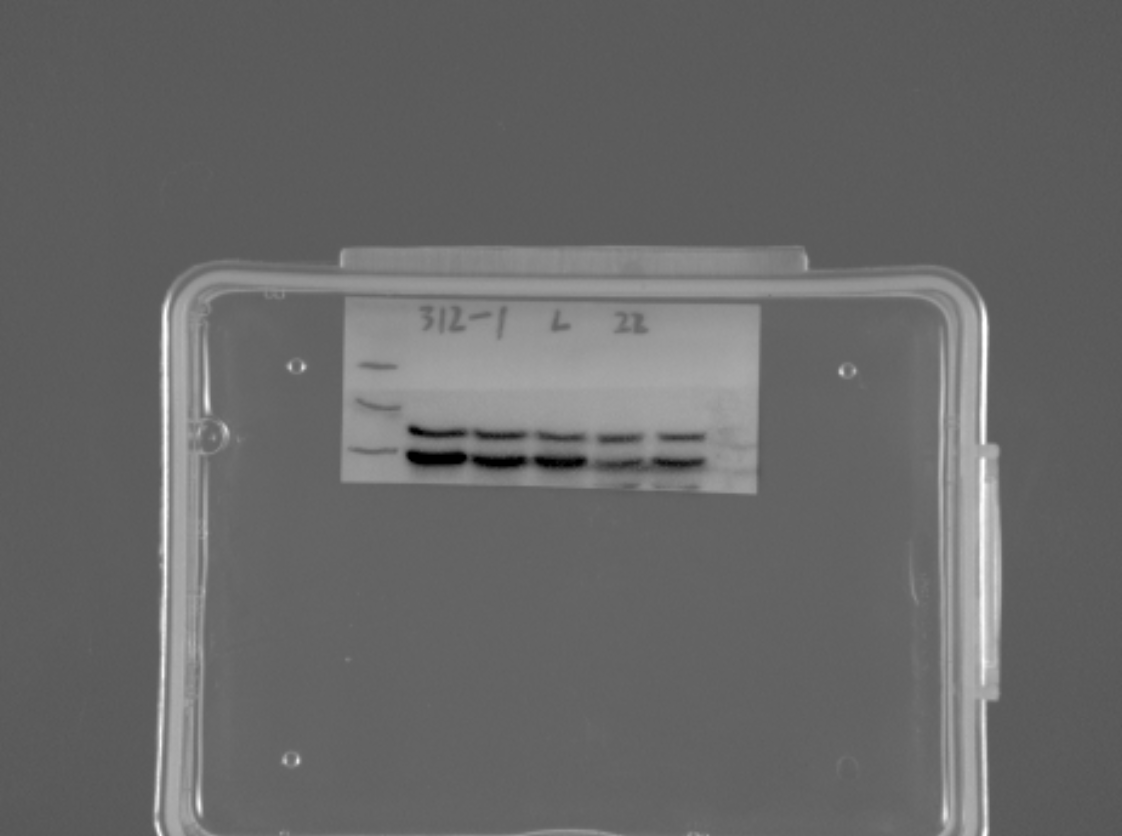

Supplement: Supplementary file 5 [file mmc5.zip › Original Images of Western blot/Fig.3/Fig.3D Caspase3.tif]

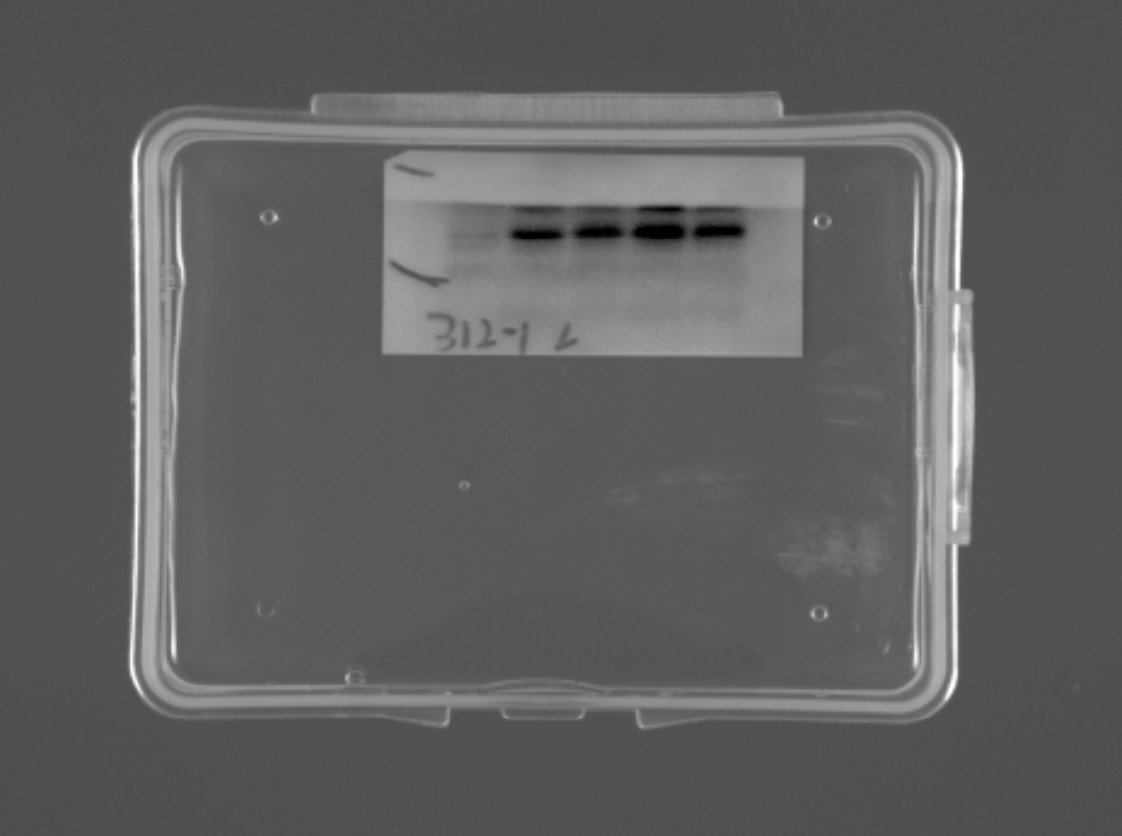

Supplement: Supplementary file 5 [file mmc5.zip › Original Images of Western blot/Fig.3/Fig.3D Cleaved-caspase3.tif]

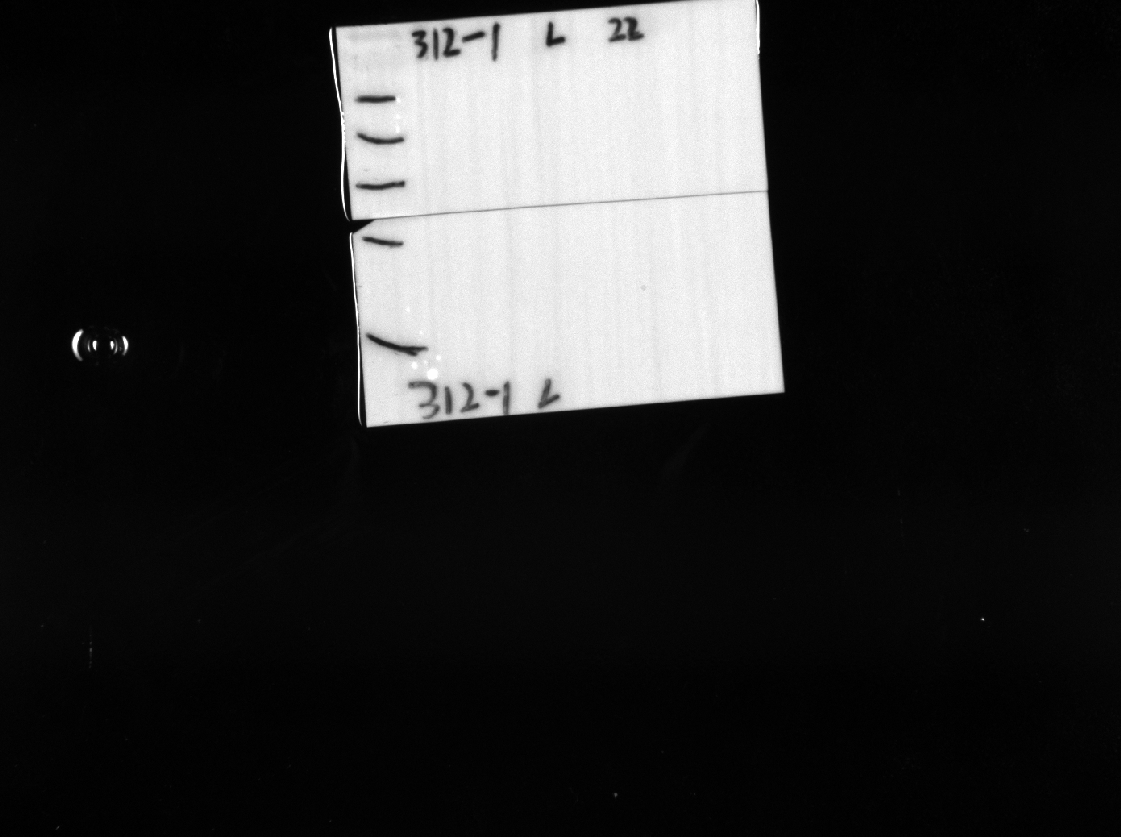

Supplement: Supplementary file 5 [file mmc5.zip › Original Images of Western blot/Fig.3/Fig.3D Full membrane.tif]

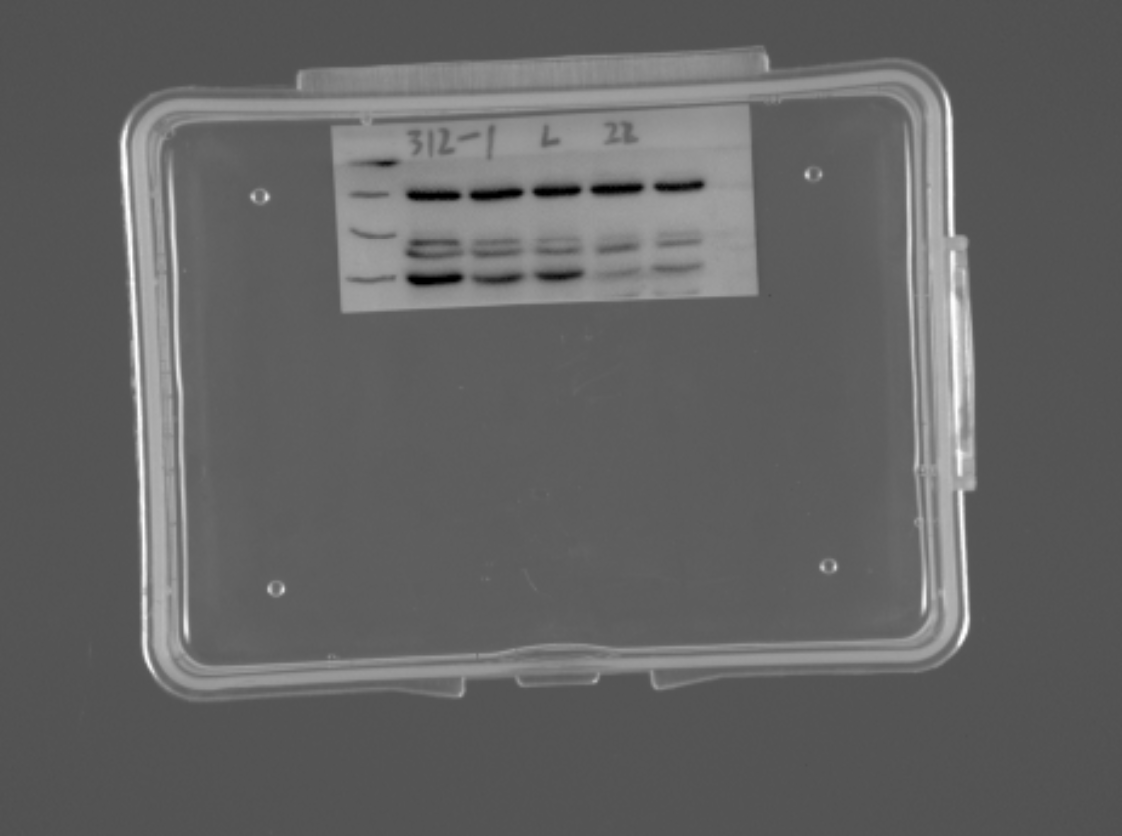

Supplement: Supplementary file 5 [file mmc5.zip › Original Images of Western blot/Fig.3/Fig.3D Tubulin.tif]

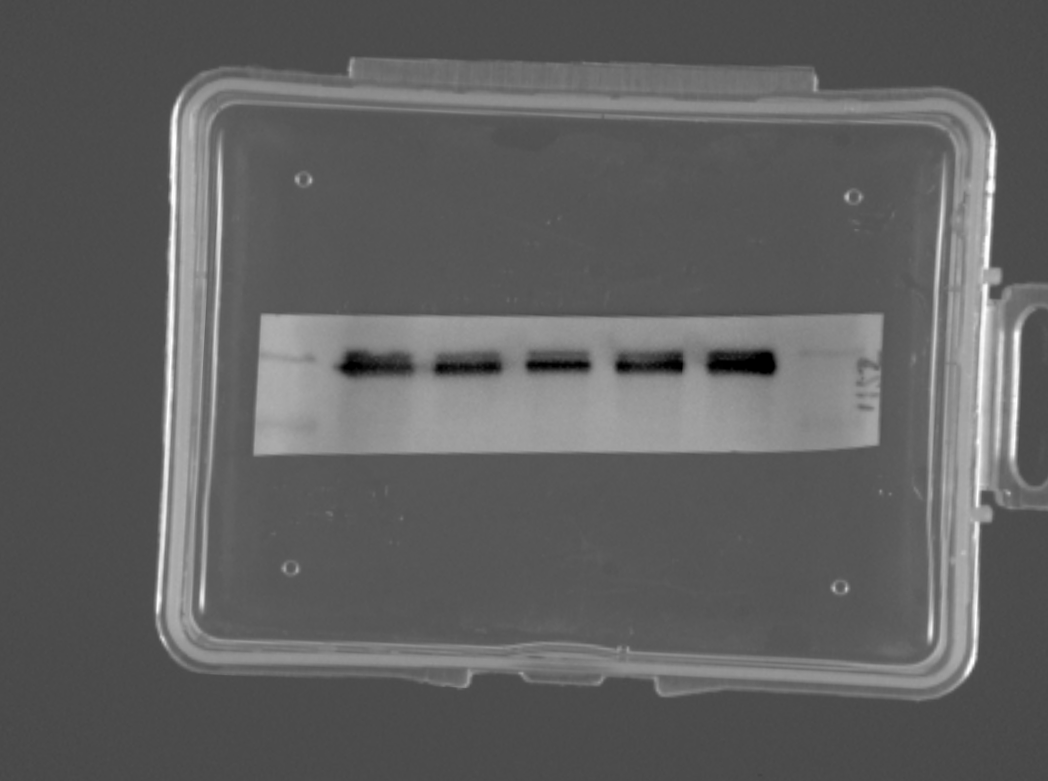

Supplement: Supplementary file 5 [file mmc5.zip › Original Images of Western blot/Fig.3/Fig.3E ERK.tif]

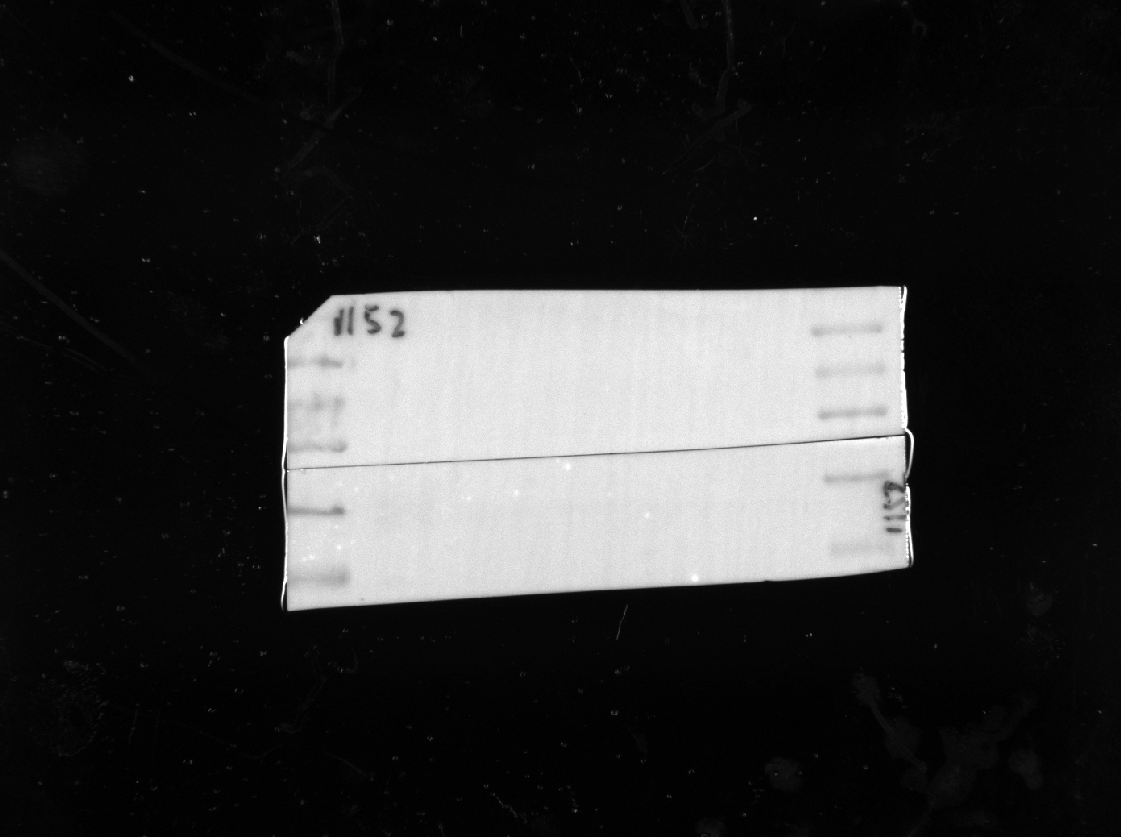

Supplement: Supplementary file 5 [file mmc5.zip › Original Images of Western blot/Fig.3/Fig.3E Full membrane.tif]

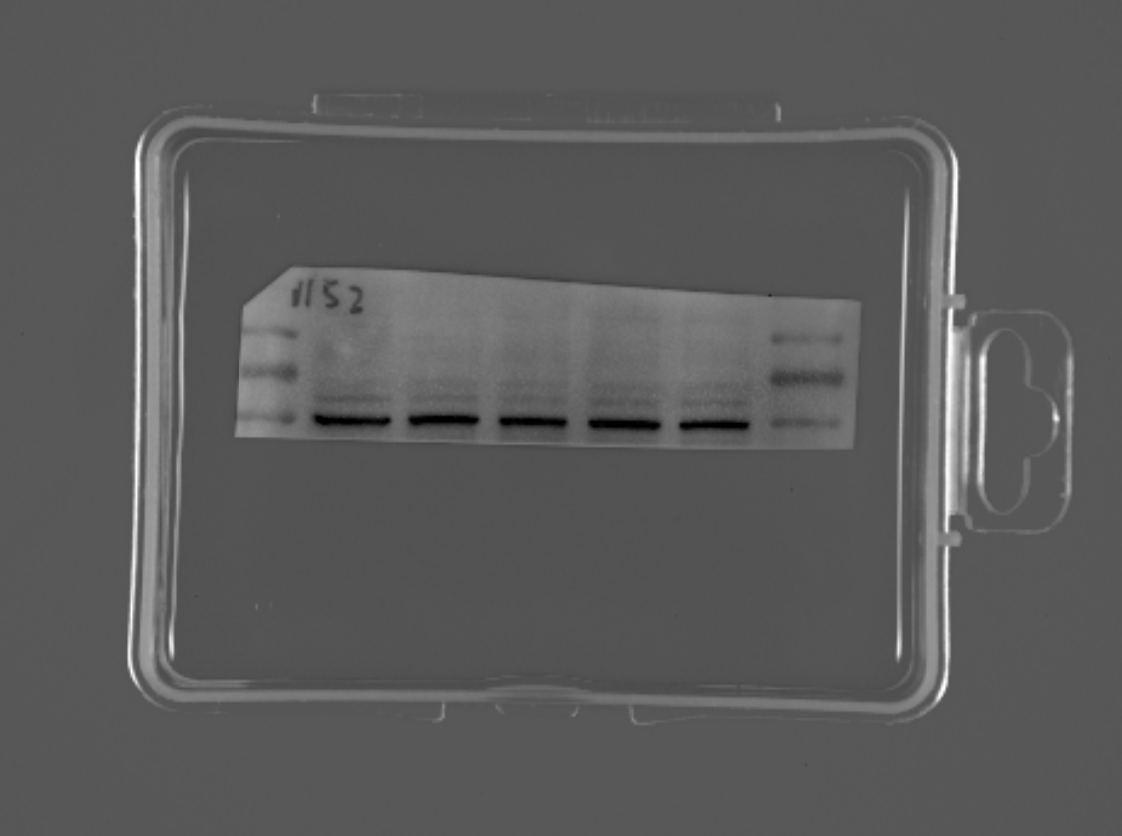

Supplement: Supplementary file 5 [file mmc5.zip › Original Images of Western blot/Fig.3/Fig.3E Tubulin.tif]

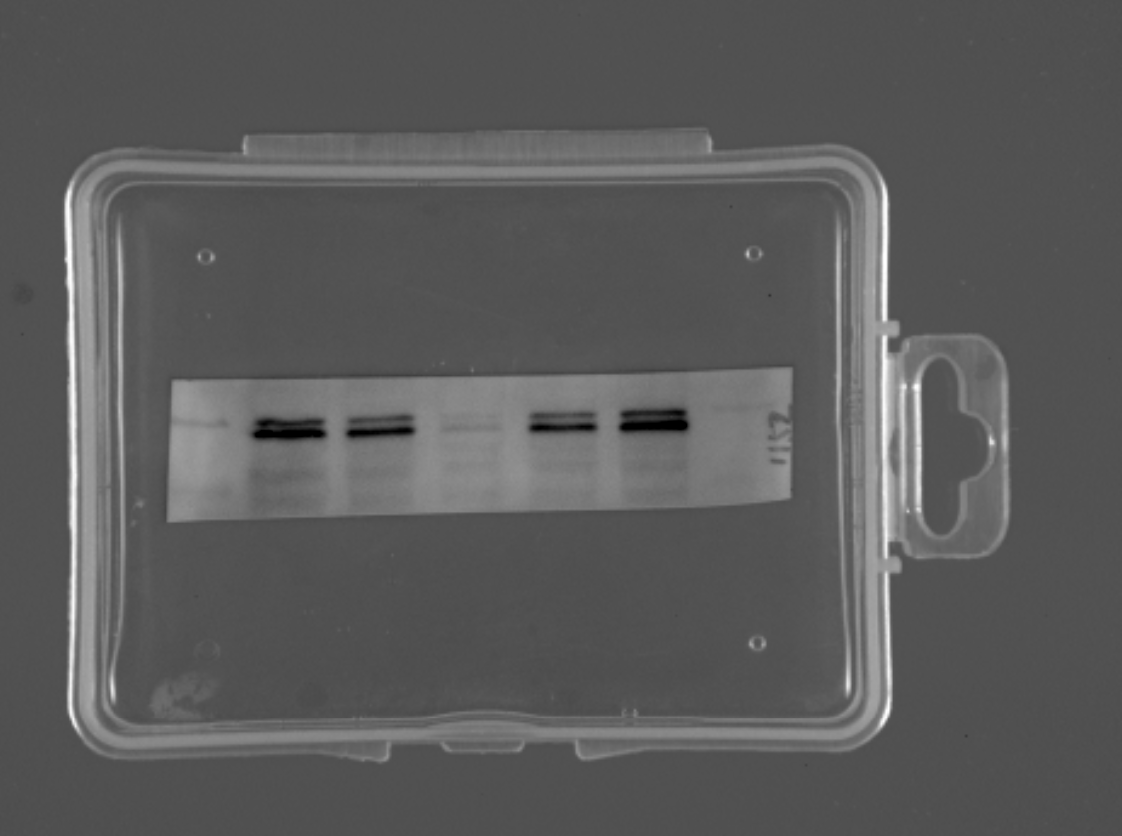

Supplement: Supplementary file 5 [file mmc5.zip › Original Images of Western blot/Fig.3/Fig.3E p-ERK.tif]

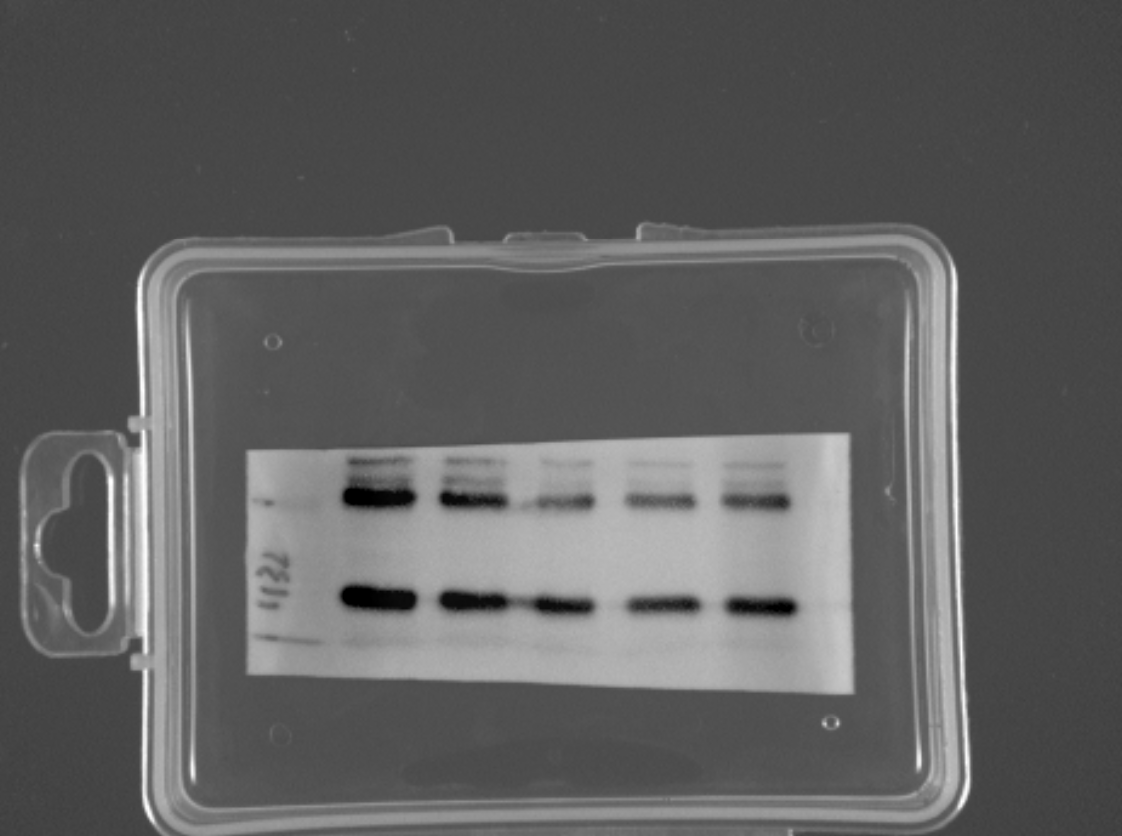

Supplement: Supplementary file 5 [file mmc5.zip › Original Images of Western blot/Fig.3/Fig.3F Bcl2 and Bax.tif]

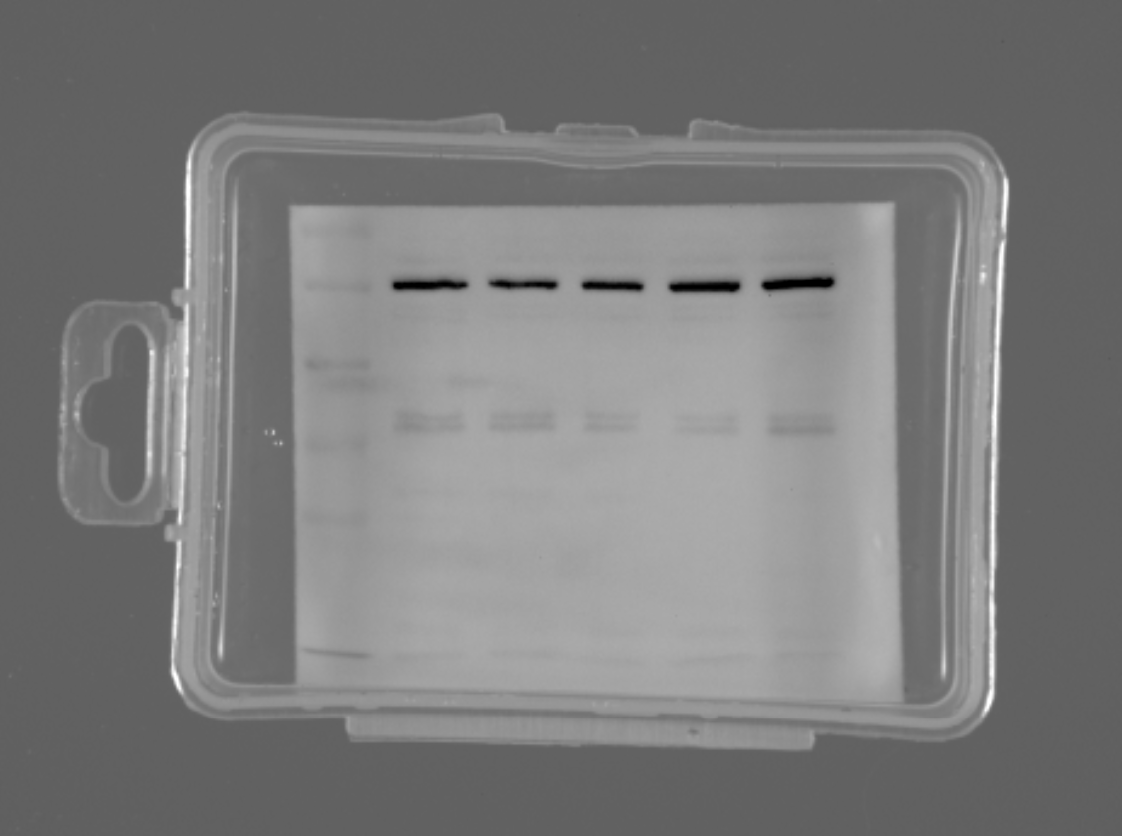

Supplement: Supplementary file 5 [file mmc5.zip › Original Images of Western blot/Fig.3/Fig.3F Tubulin.tif]

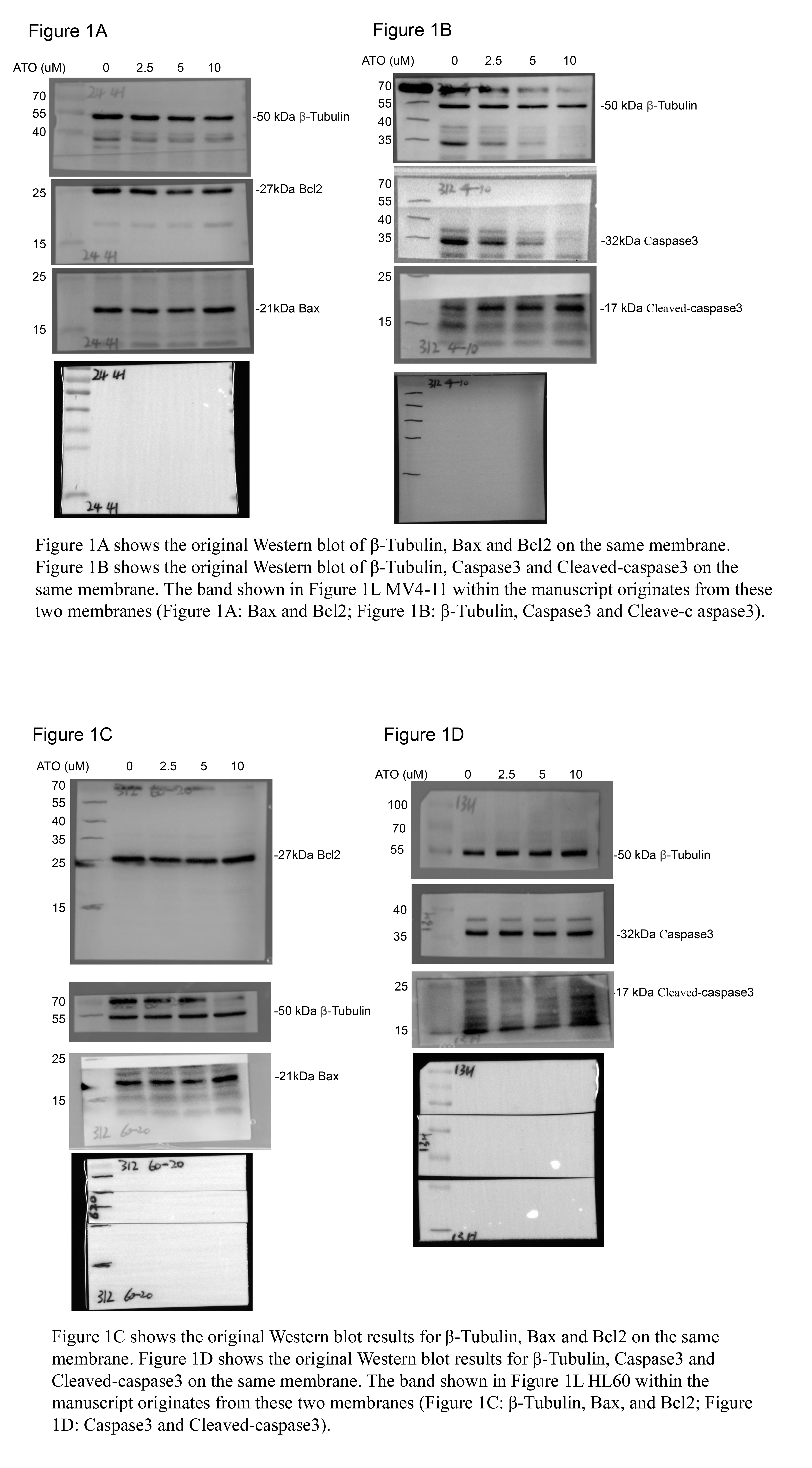

Supplement: Supplementary file 5 [file mmc5.zip › Original Images of Western blot/Figure.1.jpg]

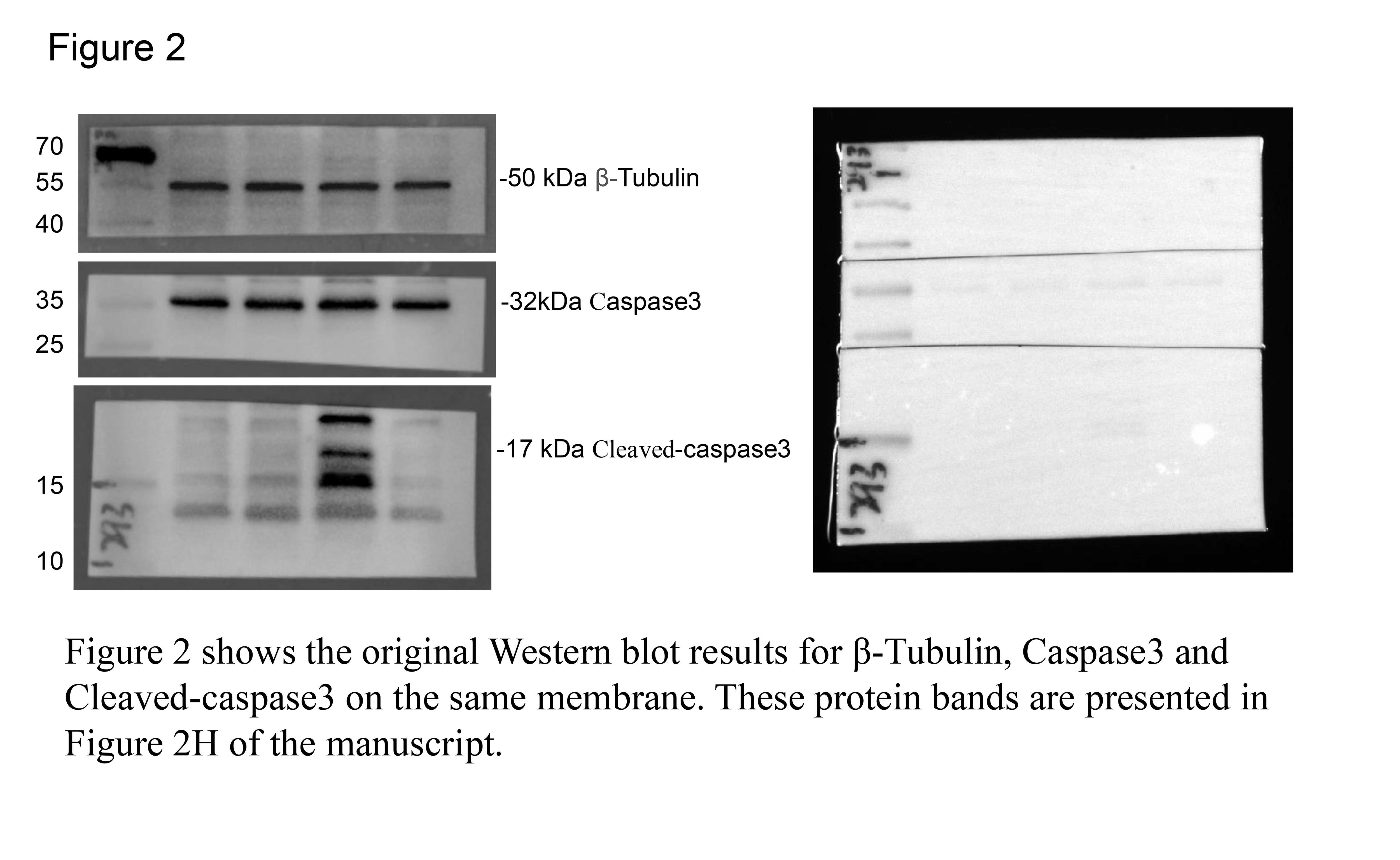

Supplement: Supplementary file 5 [file mmc5.zip › Original Images of Western blot/Figure.2.jpg]

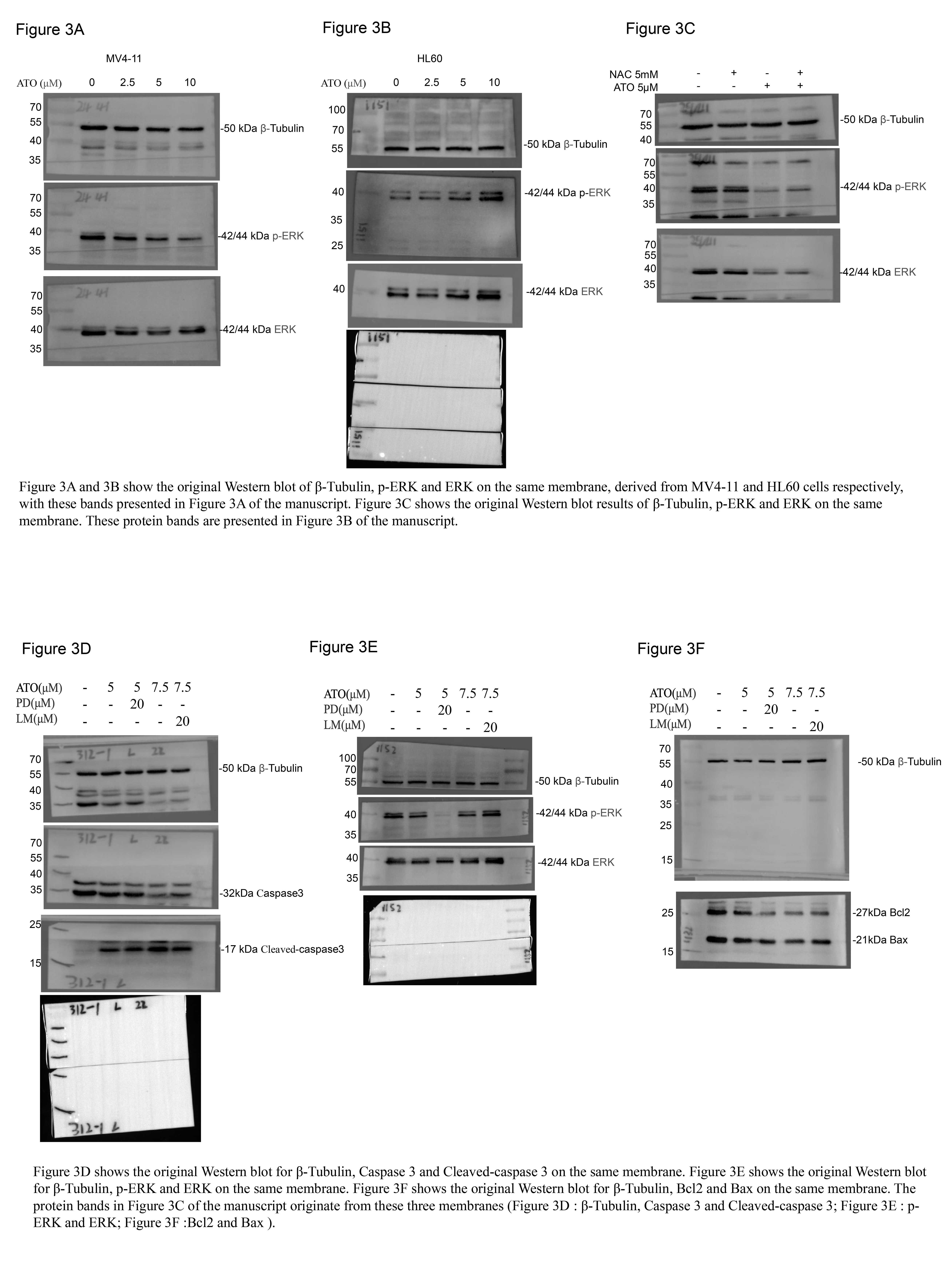

Supplement: Supplementary file 5 [file mmc5.zip › Original Images of Western blot/Figure.3.jpg]
